# Brome mosaic virus detected in Kansas wheat co-infected with other common wheat viruses

**DOI:** 10.3389/fpls.2023.1096249

**Published:** 2023-03-03

**Authors:** Nar B. Ranabhat, John P. Fellers, Myron A. Bruce, Jessica L. Shoup Rupp

**Affiliations:** ^1^ Department of Plant Pathology, Throckmorton Plant Science Center, Kansas State University, Manhattan, KS, United States; ^2^ USDA-ARS, Hard Winter Wheat Genetics Research Unit, Manhattan, KS, United States

**Keywords:** wheat virus, virome, brome mosaic virus, mixed infection, selection pressure

## Abstract

Wheat breeders are developing new virus-resistant varieties; however, it is assumed that only a few viruses or well-known viruses are present in the field. New sequencing technology is allowing for better determination of natural field virus populations. For three years, 2019-2021, Kansas wheat field surveys were conducted to determine the constituents of natural field virus populations using nanopore sequencing. During analysis, brome mosaic virus (BMV) was identified for the first time in Kansas but was in association with other wheat viruses. Brome mosaic virus was identified from 29 out of 47 different Kansas counties sampled and 44% of the total samples. BMV was found co-infected with wheat streak mosaic virus (WSMV) and Triticum mosaic virus (TriMV) in 27.8% of the samples, with WSMV only (13.9%) and co-infected with WSMV + TriMV + High Plains wheat mosaic emaravirus (HPWMoV) (13.9%). RNA genomes of Kansas BMV isolates had 99.4 to 100% nucleotide and amino acid sequence identity, respectively, to each other. RNA2a possessed relatively high divergence (π = 0.01) compared to RNA1a and RNA3a (π = 0.004). Coding regions of all BMV RNAs were considered negative for purifying selection pressure as nonsynonymous and synonymous nucleotide ratio was less than one (dNs/dS >1). The identification of BMV in Kansas virus populations adds another layer of complexity to plant breeding. This work provides information to improve tools to aid in monitoring, detecting, and determining the variation within BMV.

## Introduction

Brome mosaic virus (BMV, Genus: *Bromovirus*, family: *Bromoviridae*) is the type member of a group of icosahedral, positive-strand ssRNA viruses with a tripartite linear genome. The genome is comprised of RNA1, RNA2, and RNA3 (Ahlquist et al., 1984; Kao and Sivakumaran, 2000). RNA1 encodes protein 1a, containing capping and RNA helicase activities, RNA2 encodes protein 2a, a putative RNA-dependent RNA polymerase, and RNA3 encodes the movement protein (MP) and coat protein (CP). The CP is coded as a sub-genomic strand within RNA3 and recognized as RNA4 (Kao and Sivakumaran, 2000; Rao, 2006). BMV virions encapsidate RNA1 and RNA2 separately, whereas RNA3 is encapsidated with sub-genomic RNA4 in a single virion (Rao, 2006). The capsid of all three particles contains 180 CP subunits arranged in icosahedral symmetry (Lucas et al., 2002).

BMV is distributed worldwide as it has been reported in the United States (Mian et al., 2005; Srivatsavai, 2005; Hodge et al., 2019), Canada (Díaz-Cruz et al., 2018), South Africa (Von Wechmar and Rybicki, 1985), Estonia (Sõmera et al., 2016), Poland (Trzmiel et al., 2015), Lithuania (Urbanavičienė and Žižytė, 2012), Serbia (Tošič, 1971), Hungary (Pocsai et al., 1991), Great Britain (Gibson and Kenten, 1978), Brazil (Caetano et al., 1990), and Russia (Lane, 1974). BMV has a wide host range and mainly infects grasses of the Poaceae family, including major crops such as wheat, barley, oats, corn, and sorghum, but can infect dicot plants including soybean, common beans, faba beans, cowpea, tobacco, and *Nicotiana benthamiana* or *Chenopodium* species (Lane, 1974; Kao and Sivakumaran, 2000; Trzmiel et al., 2016; Hodge et al., 2019). Viral induced symptoms vary by plant species. In grasses, the distinct BMV symptoms include yellow mosaic, with light and dark green streaks and chlorosis similar to the symptoms caused by many cereal viruses (Slykhuis, 1976; Mian et al., 2005; He et al., 2021).

The primary transmission route of BMV in plants is still ambiguous (He et al., 2021), but spread has been reported by several modes of transmission. Mechanical inoculation is highly efficient as the virus can be transmitted by farm machinery in the field (Lane, 1974; Mian et al., 2005) and by using BMV-infected plant sap, purified virions or infectious clones (Srivatsavai, 2005; Hodge et al., 2019; He et al., 2021). A low rate of transmission by vectors, including flea beetle (*Altica foliaceae*, (Srivatsavai, 2005), Russian wheat aphid (*Diuraphis noxia*), nematodes (*Longidorus breviannulatus* and *Xiphinema* spp., (Schmidt et al., 1963; Huff et al., 1987), and bird cherry-oat aphid (*Rhopalosiphum padi*) (Rybicki and Von Wechmar, 1982; Damsteegt et al., 1992) were recorded in greenhouse experiments with wheat or barley as a host. BMV transmission was also associated with wheat stem rust (*Puccinia graminis tritici*) as BMV virus particles were attached to the uredospores surface collected from fields (Erasmus et al., 1983). Infectious BMV was detected in water resources surrounding cereals fields, demonstrating that the virus can survive without its host and vector (Jeżewska et al., 2019).

BMV was studied as a model for RNA virus biology and as an expression vector in recombinant DNA technology (Kao and Sivakumaran, 2000; He et al., 2021), however, only a few studies have been conducted to evaluate the BMV incidence on economically important crops. BMV was the dominant virus in wheat fields in Hungary in, 1994-95 (Papp et al., 1996), and an average 13% BMV incidence in Alabama wheat field samples collected in, 2004 (Srivatsavai, 2005). Greenhouse studies showed that BMV reduced wheat height, weight, and yields (Pocsai et al., 1991). Hodge et al., 2019 reported up to 61% yield loss on soft red winter wheat when inoculated at early growth stages. BMV was detected with a high prevalence in wheat and showed a potentially high risk to wheat production in Ohio (Hodge et al., 2020). Mixed infection of multiple wheat viruses in a single plant compounds the risk, resulting in a synergistic yield reduction (Lane, 1974).

As Kansas is one of the top wheat-producing states in the USA (USDA, National Agricultural Statistics Service, 2021), any new threat to wheat production could lead to millions of dollars in lost productivity (Hollandbeck et al., 2021). Multiple virus infections in a single plant are common. Frequent monitoring of viral pathogens and accurate diagnosis of field virus diversity is essential to design management strategies. There is a great demand for accurate new techniques to identify multiple cereal virus infections in a single wheat plant. This study presents the detection and characterization of BMV isolates obtained from Kansas wheat using Oxford Nanopore sequencing techniques (ONT). ONT has proven to be a powerful method of detecting new plant viruses, specifically those in wheat (Fellers et al., 2019; Dong et al., 2022). The information regarding BMV co-infection with other viruses provides the foundation for accurate diagnosis in mixed infection of multiple viruses and the use of ONT for the dual purpose of surveillance and in-depth genetic and evolutionary characterization.

## Materials and methods

### Field survey and RNA extraction

An ad-hoc field survey was conducted during the wheat growing season from May to July, from, 2019 to, 2021 in major wheat growing counties of Kansas. A total of 84 samples exhibiting yellow discoloration or mosaic patterns were collected from the newest wheat leaf taken between jointing to the soft dough stage from 47 different counties of Kansas. Leaf tissue of each sample was stored at - 20°C until the tissue could be processed for RNA extraction. Total RNA was extracted using the *mir*Vana RNA extraction kit (Ambion Catalog number: AM1560, Thermo Fisher Scientific, MA, USA) from 200 mg of tissue following the company’s instructions. RNA concentration was measured by NanoDrop spectrophotometer (NanoDrop Technologies, Rockland, DE, USA). Only samples with concentrations ranging from 100-120 ng/ul and 260/280 values between 1.8-2.0 were used in library preparation. Seven µg of total RNA was treated with 1 µl of DNase using Turbo DNase-Free ™ kit (AM, 1907, Ambion^®^, Thermo Fisher, MA, USA) in a 50 µl reaction volume according to the manufacturer’s instruction.

### Nanopore sequencing

Samples were barcoded using the Oxford PCR-cDNA Barcoding kit (SQK-PCB109) during preparation of the MinION cDNA library following the manufacturer’s instruction (Oxford Nanopore Technologies, Oxford, U.K.) with the following modifications. A total reaction volume of 11 µl was prepared with 1µl (100 to 120 ng/µl) total RNA, 1 µl of 2 µM VN primers (oligo dT VNP, SQK-PCB109, ONT), 1 µl of 10 mM dNTPs (Invitrogen, catalog number: 1875160), 8 µl of nuclease-free water (NFW) and incubated at 65°C for 5 minutes. Second strands were synthesized by mixing 4 µl 5x RT buffer (Invitrogen, catalog number, 18090200), 1 µl of RNaseOUT (Invitrogen, catalog number, 10777019), 2 µl of 10 µM Strand-Switching primer (SSP, SQK-PCB109, ONT), and 1 µl of NFW was added to the barcoding mix and incubated at 42°C for 2 minutes. One µl of Maxima H minus Reverse Transcriptase (Invitrogen, catalog number, 18090200) was added to make a total volume of 20 µl and incubated for 90 minutes at 42°C, followed by heat inactivation for 5 minutes at 85°C.

Five ul of reverse-transcribed RNA was used along with 1.5 µl barcode primers (BP01 to BP12, SQK-PCB109, ONT) for each sample up to 12 samples, 18.5 µl of NFW, and 25 µl of 2X LongAmp Taq Master Mix (New England Biolabs, catalog number #M0287). PCR amplification consisted of 95°C for 30secs for initial denaturation, 15 cycles of 95°C for 15secs, 62°C for 15secs, 65°C for 12 minutes, and final extension of 6 minutes at 65°C. One µl of NEB exonuclease 1 (New England Biolabs, catalog number #M0293) was added after completing PCR and incubated for 15 minutes at 37°C, followed by 80°C for 15 minutes. After completing the incubation and heating, 40 µl of AMPure XP beads (Beckman Coulter, #A63881) were added to the reaction and incubated for 5 minutes in a rotator mixer at room temperature (18-20°C). Beads were washed with freshly prepared 70% ethanol following the manufacturer’s instructions (Oxford Nanopore Technologies, Oxford, U.K.). The cDNA library was eluted in 12 µl of Elution buffer (EB, SQK-PCB109, ONT). After measuring the concentration of the cDNA library, the barcoded samples were pooled to a final volume of 11 µl and a 1 µl of Rapid Adaptor (RAP, SQK-PCB109, ONT) was added. The 12 µl total volume was incubated at 22°C for 15 minutes. The prepared library was loaded on the MinION R9.4.1 flow cell (Oxford Nanopore) following the manufacturer’s priming and loading instruction (Oxford Nanopore Technologies, Oxford, U.K.).

### Bioinformatics

MinKNOW operating software (version: 21.06.13, Oxford Nanopore Technologies, Oxford, U.K.) provided the fast5 raw data. These signals were translated to nucleotide bases by using the guppy basecaller (version 5.0.11 + 2b6dbff) high accuracy option (config dna_r8.4.1_450bps_hac.cfg, (Wick et al., 2019) to get fastq data. Barcoded files were sorted into individual folders using guppy barcoder. Adapters were trimmed using porechop v0.2.3 (Wick et al., 2017). Reads were sorted for 75 bp - 30 Kbp using Nanofilt v2.3.0 (De Coster et al., 2018). Thus, obtained reads were mapped against cereal virus reference genomes. Using CLC Genomic Workbench^®^ v21.0.4 (Qiagen, MD, United States) by aligning reads against reference genome of the most common cereal viruses (**Supplementary Table 1**). The following parameters were used in CLC workbench, reads were mapped to reference using the parameters following resequencing analysis with masking mode = no masking, match score = 1, mismatch cost = 2, cost of insertions and deletions = Linear gap cost, insertion cost = 3, deletion cost = 3, length fraction = 0.5, similarity fraction = 0.8, global alignment = no, non-specific match handling = map randomly, output mode = create stand-alone read mappings, create report = yes, collect unmapped reads = no. The consensus sequences with low coverage were blasted in the NCBI nucleotide (blastn) database to confirm the presence of BMV. The minimum sequence read length of consensus sequence for the identification of a particular virus was, 1000 bp considered as lower limit to count.

### Sequence alignment, percent identity, and similarity

The coding region of each protein (RNA1a, RNA2a, MP, and CP) of BMV isolates sequenced in this study and selected isolates from GenBank (**Supplementary Table 2**) were aligned using Multiple Sequence Alignment ‘MUSCLE’ alignment in Mega X (Kumar et al., 2018) with default parameters (Max Iterations 16, Cluster Method) separately. Aligned sequences were analyzed to obtain percent identity using an ‘MUSCLE’ online program supported by EMBL-EBI (Edgar, 2004). The amino acid sequence alignments obtained from Mega X were analyzed using the SMS (Sequence Manipulation Suite) online program available through bioinformatics.org (Stothard, 2000) to obtain amino acid percent identity and similarity of the coding regions of each protein of BMV.

### Phylogenetic analysis

The cassia yellow blotch virus (CYBV) and olive latent virus 2 (OLV) were used as outgroups (**Supplementary Table 3**). To construct the cladograms, the best fitting nucleotide substitution models were determined by the maximum likelihood fits (Nei and Kumar, 2000; Kumar et al., 2018). The best-fitted model was selected based on the lowest Akaike information criterion (AIC) and Bayesian information criterion (BIC) scores (Guindon and Gascuel, 2003). These models were TN93 + G (Tamura-Nei substitution model with Gamma distributed rate) for RNA1a and RNA2a, K2 + I (Kimura-2-parameter model with Invariant sites) for MP and CP of BMV. Maximum likelihood cladograms were constructed using Mega X with parameters as follows: the number of Bootstrap replications of, 1000, nucleotide substitution model as mentioned above for coding regions of different RNAs, and the number of threads of 4.

### Population genetics analysis

The population genetic analysis was done with the BMV US isolate sequences obtained from this study and isolates with complete coding sequence available in GenBank. The program DnaSP version 5.10 (Librado and Rozas, 2009) was used to analyze the population genetic parameters including the number of segregating sites (s), the total number of mutations (η), nucleotide diversity (π), and mutation rate (θw) each were calculated on the protein-coding sequence of RNA1a (five isolates: 20SM3, 19RP1, BMV_OK, BMV_M1, and BMV_M2), of RNA2a and sub-genomic RNA4 (coat protein, CP) (seven isolates: 20SM3, 19RP1, BMV_OK, BMV_OH, BMV_OH2, BMV_M1, and BMV_M2) and for RNA3a (movement protein, MP) (12 isolates; 7 isolates obtained from this study and five BMV_OK, BMV_OH, BMV_OH2, BMV_M1, and BMV_M2). The program MEGA X (Kumar et al., 2018) was used to estimate non-synonymous substitutions (dN), synonymous substitutions (dS) and their ratio (dN/dS = ω) using the bootstrap variance estimation method with, 1000 replicates under the model of Kumar method (Kimura 2-para) for each encoded protein.

## Results

**Figure 1:**
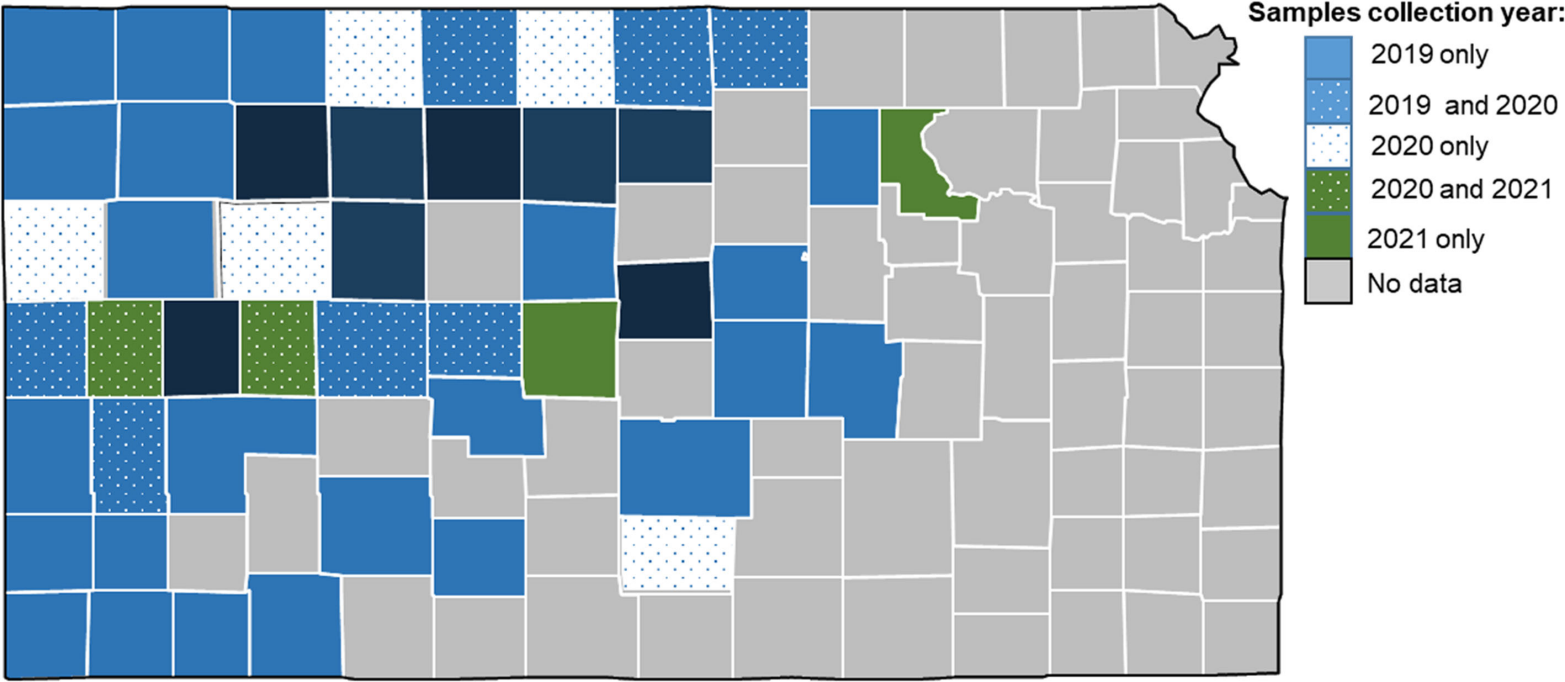
Map of Kansas counties where samples were collected in 2019, 2020, and 2021. Counties with no data indicates never sampled during this study.

Between the years of, 2019 and, 2021, 84 field samples consisting of wheat leaf tissue from plants at the jointing to soft dough stage were taken from wheat fields across the state of Kansas (**Figure 1**). Plants were selected based on expression of virus-like symptoms of mosaic, yellowing, and stunting. Total RNA was extracted, but varied in quantity and quality based on the original sample and how it was stored. Oxford nanopore based sequencing produced differing numbers of total reads ranging from 1.29 million (20SD4) to just 60,378 (20WH).

### Co-infection of brome mosaic virus

**Figure 2:**
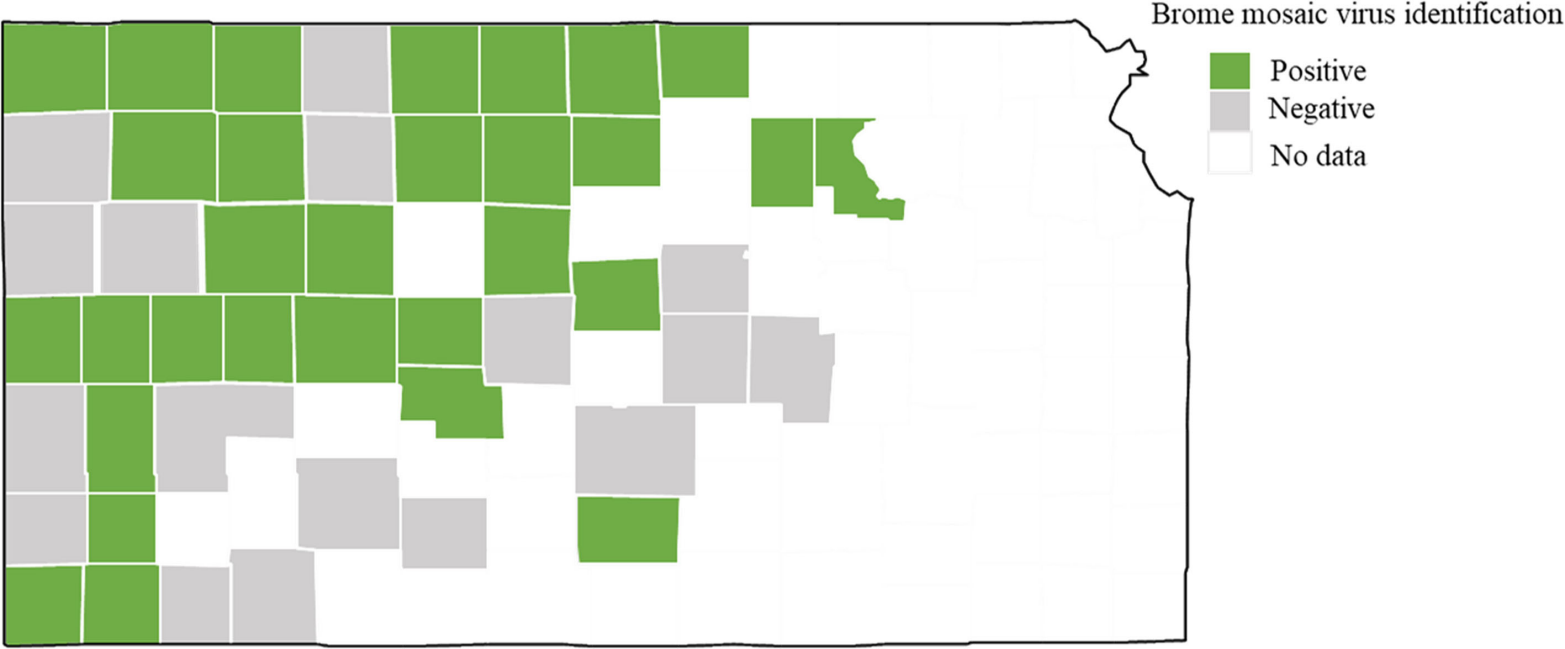
Map of Kansas with counties where brome mosaic virus was identified using Nanopore sequencing. Counties with no data indicates never sampled during this study.

**Figure 3:**
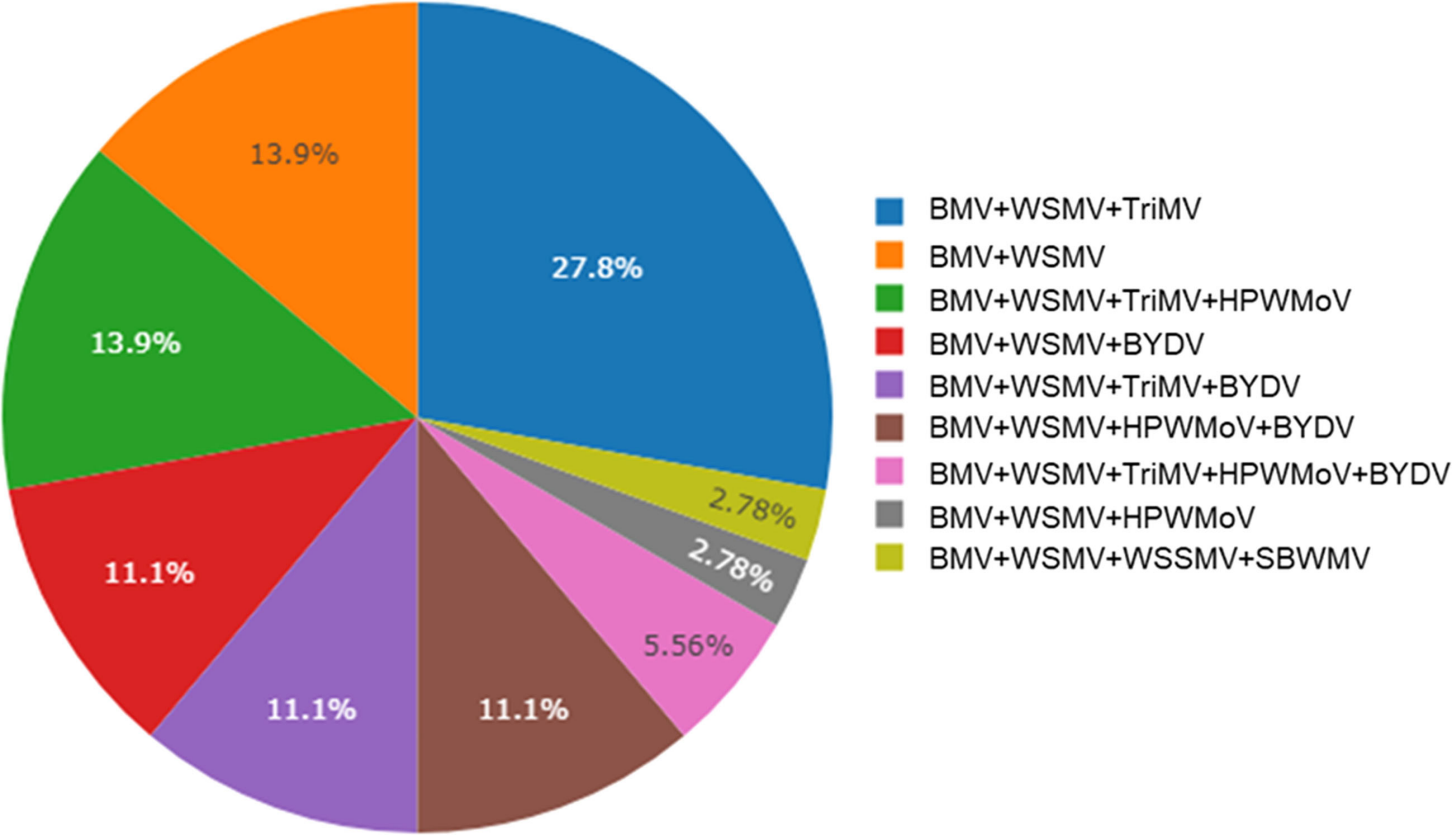
Percent incidence (n = 37) of brome mosaic virus co-infected with of wheat streak mosaic virus (WSMV), triticum mosaic virus (TriMV), High Plains wheat mosaic emaravirus (HPWMOV), brome mosaic virus (BMV), barley yellow dwarf virus (BYDV), wheat spindle streak mosaic virus (WSSMV), cereal yellow dwarf virus (CYDV), soilborne wheat mosaic virus (SBWMV)in leaf samples collected from Kansas wheat fields detected by Nanopore sequencing. Virus-like symptomatic wheat leaves were collected from winter wheat field in 2019, 2020, and 2021.

Brome mosaic virus was identified from 29 different counties of Kansas out of 47 counties sampled (**Figure 2**). 44% (37 out of 84 total samples) of the samples processed using ONT were positive for BMV. Out of these 37 positive samples, BMV was found co-infected with WSMV and TriMV (27.8%), followed by co-infected with WSMV only (13.9%) and with WSMV + TriMV + HPWMoV (13.9%) (**Figure 3**). BMV was found co-infected with BYDV+WSMV+ TriMV+ HPWMoV (11.1%). 2.8% of the BMV positive samples were identified co-infected with wheat spindle streak mosaic virus (WSSMV) and soilborne wheat mosaic virus (SBWMV) (**Figure 3**).

### Sequencing BMV genome

We obtained an average of 4.52 x 10^5^ raw reads from 37 samples obtained from Nanopore sequencing (**Supplementary Table 3**). The average reads of BMV and coverage of each sample were varied (**Supplementary Table 4**). Two complete genome and nine complete nucleoprotein sequence of total 11 isolates (**Supplementary Table 4**) were obtained and deposited in GenBank. The remaining samples with missing few nucleotides in the coding regions of the genome were excluded from further study. Complete genomes of RNA1, RNA2, and RNA3 of BMV were obtained from Smith and Republic counties of Kansas. However, the complete sequence of movement protein was obtained from Cheyenne, Decatur, Ness, and Jewell counties (**Supplementary Table 4**).

The complete coding sequences of RNA1a and RNA2a (**Table 1**, **Table 2** respectively) as well MP and CP (**Table 3**, **Table 4** respectively) were aligned with the sequences obtained from the GenBank.

**Table 1:** Nucleotides/amino acid sequence identity (similarity) of RNA1/RNA1a genome of rome mosaic virus (BMV) isolates from Kansas and other known BMV isolates retrieved from GeneBank.

BMV isolates	20SM3	19RP1	BMV-OH	BMV-OH2	BMV-OK	BMV-M1
20SM3	–					
19RP1	99.5/100 (100)	–				
BMV-OH	99.3/99.6 (99.7)	99.2/99.6 (99.7)	–			
BMV-OH2	99.3/99.7 (99.7)	99.3/99.7 (99.7)	99.7/99.8 (99.9)	–		
BMV-OK	99.5/99.8 (99.8)	99.5/99.8 (99.8)	99.2/99.5 (99.6)	99.5/99.7 (99.7)	–	
BMV-M1	99.3/99.5 (99.7)	99.1/99.5 (99.7)	98.8/99.1 (99.4)	98.8/99.2 (99.4)	99.1/99.3 (99.5)	–
BMV-M2	99.3/99.7 (99.9)	99.5/99.7 (99.9)	99.1/99.3 (99.6)	99.3/99.4 (99.6)	99.5/99.5 (99.7)	98.9/99.2 (99.6)
BMV-UK	99.5/99.7 (99.8)	99.5/99.7 (99.8)	99.2/99.3 (99.5)	99.3/99.4 (99.5)	99.5/99.5 (99.6)	99.1/99.3 (99.5)
BMV-Germany	99.6/99.7 (99.8)	99.5/99.7 (99.8)	99.2/99.3 (99.5)	99.3/99.4 (99.5)	99.6/99.5 (99.6)	99.1/99.3 (99.5)
BMV-CZ	98.2/98.9 (99.2)	97.9/98.9 (99.1)	97.7/98.5 (98.9)	97.7/98.7 (98.9)	97.8/98.8 (98.9)	98.2/99.1 (99.3)
BMV-Estonia	98.5/99.5 (99.5)	98.2/99.2 (99.2)	98.1/99.1 (99.2)	98.2/99.2 (99.2)	98.1/99.3 (99.3)	98.3/99.3 (99.6)
	BMV_M2	BMV_UK	BMV_Germany	BMV_CZ		
BMV_UK	99.4/99.4 (99.7)	–				
BMV_Germany	99.5/99.4 (99.7)	99.9/100 (100)	–			
BMV_CZ	97.6/98.7 (99.1)	97.9/98.8 (98.9)	97.9/98.8 (98.9)	–		
BMV_Estonia	98.1/99.2 (99.4)	98.2/99.3 (99.3)	98.3/99.3 (99.3)	97.8/99.2 (99.5)		

The nucleotide percentage identity was calculated from ClustalW alignment online tools (Clustal 2.1) (Madeira et al., 2019), and amino acid sequence identity and similarity were calculated from a sequence manipulation suite (Stothard, 2000).

**Table 2:** Nucleotides/amino acid sequence identity (similarity) of RNA2/RNA2a genome of brome mosaic virus (BMV) isolates from Kansas and other known BMV isolates retrieved from GeneBank.

BMV isolates	20SM3	19RP1	BMV_OH	BMV_OH2	BMV_OK	BMV_M1
20SM3	–					
19RP1	99.6/100 (100)	–				
BMV-OH	99.2/99.4 (99.6)	99.3/99.9 (99.9)	–			
BMV-OH2	99.4/99.9 (99.9)	99.5/99.9 (99.9)	99.9/100 (100)	–		
BMV-OK	99.2/99.4 (99.6)	99.3/99.4 (99.6)	99.1/99.5 (99.8)	99.2/99.5 (99.8)	–	
BMV-M1	98.3/99.0 (99.5)	98.1/99.0 (99.5)	97.9/99.2 (99.6)	98.0/99.2 (99.6)	97.9/98.7 (99.4)	–
BMV-M2	99.3/99.6 (99.8)	99.3/99.6 (99.8)	99.2/99.8 (99.9)	99.3/99.8 (99.9)	99.2/99.5 (99.9)	98.1/98.9 (99.5)
BMV-UK	98.3/99.5 (99.8)	99.5/99.5 (99.8)	99.3/99.6 (99.9)	99.4/99.6 (99.9)	99.2/99.2 (99.6)	98.1/98.8 (99.5)
BMV-Germany	99.4/99.6 (99.8)	99.6/99.6 (99.8)	99.5/99.8 (99.9)	99.6/99.8 (99.9)	99.3/99.3 (99.6)	98.2/98.9 (99.5)
BMV-CZ	98.4/98.8 (99.5)	98.2/98.8 (99.5)	98.0/98.9 (99.6)	98.1/98.9 (99.6)	98.0/98.4 (99.4)	98.3/99.1 (99.8)
BMV-Estonia	97.5/98.5 (99.0)	97.4/98.5 (99.1)	97.2/98.7 (99.2)	97.3/98.7 (99.2)	97.2/98.2 (98.9)	97.5/98.8 (99.3)
	BMV_M2	BMV_UK	BMV_Germany	BMV_CZ		
BMV_UK	99.2/99.4 (99.8)	–				
BMV_Germany	99.4/99.5 (99.8)	99.9/99.9 (100)	–			
BMV_CZ	98.2/98.8 (99.5)	98.1/98.7 (99.5)	98.3/98.7 (99.5)	–		
BMV_Estonia	97.3/98.3 (99.0)	97.4/98.4 (99.0)	97.4/98.4 (99.0)	97.2/98.5 (99.3)		

The nucleotide percentage identity was calculated from ClustalW alignment online tools (Clustal 2.1) (Madeira et al., 2019), and amino acid sequence identity and similarity were calculated from a sequence manipulation suite (Stothard, 2000).

**Table 3:** Nucleotides/amino acid sequence identity (similarity) of RNA3/RNA3a (Movement protein, MP) genome of brome mosaic virus (BMV) isolates from Kansas and other known BMV isolates retrieved from GeneBank.

BMV isolates	20SM3	19CN1	19CN3	19DC1	19NS2	19JW1
19CN1	100/100 (100)	–				
19CN3	100/100 (100)	100/100 (100)	–			
19DC1	100/100 (100	100/100 (100)	100/100 (100)	–		
19NS2	99.8/99.7 (100)	99.8/99.7 (100)	99.8/99.7 (100)	99.8/99.7 (100)	–	
19JW1	99.8/99.7 (99.7)	99.8/99.7 (99.7)	99.8/99.7 (99.7)	99.8/99.7 (99.7)	99.8/99.3 (99.7)	–
19RP1	99.8/99.7 (99.7)	99.8/99.7 (99.7)	99.8/99.7 (99.7)	99.8/99.7 (99.7)	99.8/99.3 (99.7)	100/100 (100)
BMV-OH2	99.3/99.3 (100)	99.3/99.3 (100)	99.3/99.3 (100)	99.3/99.3 (100)	99.3/99.0 (100)	99.3/99.0 (99.7)
BMV-OH	99.5/99.3 (100)	99.5/99.3 (100)	99.5/99.3 (100)	99.5/99.3 (100)	99.5/99.0 (100)	99.5/99.0 (99.7)
BMV_M1	99.3/99.7 (99.7)	99.3/99.7 (99.7)	99.3/99.7 (99.7)	99.3/99.7 (99.7)	99.3/99.3 (99.7)	99.3/99.3 (99.3)
BMV-M2	99.6/99.0 (99.7)	99.6/99.0 (99.7)	99.6/99.0 (99.7)	99.6/99.0 (99.7)	99.6/98.7 (99.7)	99.6/98.7 (99.3)
BMV-OK	99.8/99.7 (100)	99.8/99.7 (100)	99.8/99.7 (100)	99.8/99.7 (100)	99.8/99.3 (100)	99.8/99.3 (99.7)
BMV-UK	99.6/99.0 (99.3)	99.6/99.0 (99.3)	99.6/99.0 (99.3)	99.6/99.0 (99.3)	99.7/99.0 (99.3)	99.6/98.7 (99.0)
BMV-Germany	99.8/99.7 (99.7)	99.8/99.7 (99.7)	99.8/99.7 (99.7)	99.8/99.7 (99.7)	99.8/99.7 (99.7)	99.8/99.3 (99.3)
BMV-Estonia	99.6/100 (100)	99.6/100 (100)	99.6/100 (100)	99.6/100 (100)	99.3/99.7 (100)	99.3/99.7 (99.7)
BMV-CZ	98.6/98.4 (98.7)	99.6/98.4 (98.7)	98.6/98.4 (98.7)	99.6/98.4 (98.7)	98.4/98.0 (98.7)	98.4/98.4 (98.7)
	19RP1	BMV-OH2	BMV_OH	BMV-M1	BMV-M2	BMV-OK
BMV-OH2	99.3/99.0 (99.7)	–				
BMV-OH	99.5/99.0 (99.7)	99.9/100 (100)	–			
BMV_M1	99.3/99.3 (99.3)	98.9/99.0 (99.7)	99.0/99.0 (99.7)	–		
BMV-M2	99.6/98.7 (99.3)	99.3/99.0 (99.7)	99.5/99.0 (99.7)	99.1/98.7 (99.3)	–	
BMV-OK	99.8/99.3 (99.7)	99.6/99.7 (100)	99.7/99.7 (100)	99.3/99.3 (99.7)	99.8/99.3 (99.7)	–
BMV-UK	99.6/98.7 (99.0)	99.1/98.4 (99.3)	99.2/98.4 (99.3)	99.3/98.3 (99.3)	99.3/98.0 (99.0)	99.6/98.7 (99.3)
BMV-Germany	99.8/99.3 (99.3)	99.3/99.0 (99.7)	99.5/99.0 (99.7)	99.3/99.3 (99.3)	99.6/98.7 (99.3)	99.8/99.3 (99.7)
BMV-Estonia	99.3/99.7 (99.7)	98.9/99.3 (100)	99.0/99.3 (100)	99.1/99.7 (99.7)	99.1/99.0 (99.7)	99.3/99.7 (100)
BMV-CZ	98.4/98.4 (98.7)	97.9/97.7 (98.7)	98.0/97.7 (98.7)	98.1/98.0 (98.4)	98.1/97.4 (98.4)	98.4/98.0 (98.7)
	BMV_UK	BMV_Germany	BMV_Estonia			
BMV_Germany	99.8/99.3 (99.7)	–				
BMV_Estonia	99.1/99.0 (99.3)	99.3/99.7 (99.7)	–			
BMV_CZ	98.1/97.4 (98.0)	98.4/98.0 (98.4)	98.8/98.4 (98.7)			

The nucleotide percentage identity was calculated from ClustalW alignment online tools (Clustal 2.1) (Madeira et al., 2019), and amino acid sequence identity and similarity were calculated from a sequence manipulation suite (Stothard, 2000).

**Table 4:** Nucleotides/amino acid sequence identity (similarity) of RNA4 (coat protein, CP) genome of brome mosaic virus (BMV) isolates from Kansas and other known BMV isolates retrieved from GeneBank.

BMV isolates	20SM3	19RP1	BMV-OH	BMV-OH2	BMV-OK	BMV-M1
19RP1	100/100 (100)	–				
BMV-OH	100/100 (100)	100/100 (100)	–			
BMV-OH2	99.8/100 (100)	99.8/100 (100	99.8/100 (100)	–		
BMV-OK	99.6/98.9 (98.9)	99.6/98.9 (98.9)	99.7/98.9 (98.9)	99.5/98.9 (98.9)	–	
BMV-M1	98.9/99.5 (99.5)	98.9/99.5 (99.5)	98.9/99.5 (99.5)	99.8/99.5 (99.5)	98.6/98.4 (98.4)	–
BMV-M2	99.5/99.5 (99.5)	99.5/99.5 (99.5)	99.5/99.5 (99.5)	99.3/99.5 (99.5)	99.1/98.4 (98.4)	98.4/98.9 (98.9)
BMV-UK	98.9/98.4 (99.5)	98.9/98.4 (99.5)	98.9/98.4 (99.5)	98.8/98.4 (99.5)	98.8/98.4 (98.4)	98.3/97.9 (98.9)
BMV-Germany	99.6/100 (100)	99.6/100 (100)	99.6/100 (100)	99.5/100 (100)	99.3/98.9 (98.9)	98.9/99.5 (99.5)
BMV-CZ	96.8/97.4 (98.9)	96.8/97.4 (98.9)	96.8/97.4 (98.9)	96.7/97.4 (98.9)	96.7/96.8 (97.9)	96.5/96.8 (98.4)
BMV-Estonia	98.9/100 (100)	98.9/100 (100)	98.9/100 (100)	98.8/100 (100)	98.6/98.9 (98.9)	98.6/99.5 (99.5)
	BMV_M2	BMV_UK	BMV_Germany	BMV_CZ		
BMV_UK	98.4/97.9 (98.9)	–				
BMV_Germany	99.1/99.5 (99.5)	99.3/98.4 (99.5)	–			
BMV_CZ	96.3/97.4 (98.4)	95.8/96.3 (98.4)	96.5/97.4 (98.9)	–		
BMV_Estonia	98.4/99.5 (99.5)	97.9/98.4 (99.5)	98.6/100 (100)	96.8/97.4 (98.9)		

The nucleotide percentage identity was calculated from ClustalW alignment online tools (Clustal 2.1) (Madeira et al., 2019), and amino acid sequence identity and similarity were calculated from a sequence manipulation suite (Stothard, 2000).

RNA1: RNA1 of BMV encodes for methyltransferase and helicase. The nucleotide sequences of RNA1a (ORF1a) were > 99% identical and 100% amino acid sequence identity between the isolates 20SM3 and 19RP1 from Smith and Republic counties (**Table 1**). The nucleotide sequence and amino acid sequence of both isolates were > 99% identical with the other US isolates from OH, OK, and WI. However, the nucleotide sequence of BMV isolates from the Czech Republic (BMV_CZ) and Estonia (BMV_Estonia) were 98.2% and 98.5% identical with 20SM4 and were 97.9 and 98.2 identical with 19RP1 respectively. The nucleotide sequence of the BMV_CZ isolate was 97.8% to 98.3% identical with other US isolates (OH, OK, and WI).

In ORF1a, the amino acid substitutions were unique among isolates. Two Ohio isolates (BMV_OH, and BMV_OH2) share two amino acid substitutions (Q278R and D569A) out of three. BMV_OH2 and BMV_OK shared one amino acid substitution (K536I). The isolate from Estonia had eight amino acid substitutions out of 10 isolates compared (**Supplementary Figure 1**), including three consecutive amino acid substitutions and deletion from 21 to 23 (T21H, T22del, and N23H). It also shared three amino acid substitutions (A257T, D573G, and K827Q) with the BMV_CZ isolate. Both Czech and Estonian isolates showed higher variability than US isolates and isolates from UK and Germany in RNA1a.

RNA2: RNA2 of BMV encodes RNA-dependent RNA polymerase. The complete genome coding sequence of RNA2 was 99.6% identical and 100% amino acid sequence identity between the two isolates 20SM3 and 19RP1 (**Table 2**). The nucleotide sequence and amino acid sequence of both isolates were > 99% identical with other US isolates. However, the nucleotide sequence of both isolates was about 98% and 97.5% identical to the isolate from the Czech Republic and Estonia respectively. Two isolates from OH were 99.9% nucleotide and 100% amino acid sequence identical to each other. Notably, the 20SH3 and 19RP1 isolates share one amino acid substitution (E667K) in ORF2a or RNA2a which is unique to only these Kansas isolates (**Supplementary Figure 2**). Among other US isolates, there were four amino acid substitutions in BMV_OK isolate (L606W, D627E, T717M, K776R), three amino acid substitutions in BMV_M1 isolate (I609V, M655T, and l746I), two amino acid substitutions in BMV_M2 isolate (K567R and T717M), and BMV_UK isolate also had three amino acid substitutions (S132T, R277K, and T784A) (**Supplementary Figure 2**). There were six amino acid substitutions in the Czech isolate (H199R, R277K, K281R, K621R, L766S, and L809V) and five amino acid substitutions in Estonian isolate (A134V, A135D, D148E, D162V, and A677S).

RNA3: RNA3 of BMV encodes two proteins: the movement protein (MP) and the coat protein (CP). The nucleotide and amino acid sequences were 100% identical for ORF 3a (MP) among the three isolates obtained in this study (20SM3, 19CN1, and 19CN3). 19JW1 and 19 RP1 were also 100% identical for nucleotide and amino acid sequences to each other. 19NS2 was >99% nucleotide sequence and 100% amino acid sequence identity for ORF 3a with 20SM3, 19CN1, 19CN3, and 19DC1 (**Table 3**). The nucleotide and amino acid sequences of isolates 19JW1 were >99% identical for ORF 3a (MP) among isolates obtained in this study. The nucleotide sequence and amino acid sequence identity were >99% among isolates from OH, OK, WI, and Estonia. However, nucleotide sequence and amino acid sequence of ORF 3a of the Czech isolate were >98% identical among all isolates analyzed except BMV_OH2 isolate (>97% identity). The 19RP1 and 19JW1 isolates share one amino acid substitution (L275F) with each other. The isolates 19NS2 has one amino acid substation (D166N) (**Supplementary Figure 3**). BMV_OH, BMV_OH2, BMV_OK, and BMV_M2 shared one amino acid substitution (T299S). Two OH isolates of BMV shared one more isolate (V162I). BMV_M1 and BMV_UK isolates shared one amino acid substitution (P81S) and BMV_UK also shared one more amino acid substitution (D166H) to BMV_Germany. The Czech isolate has five amino acid substitutions on S57P, I99V, Q225R, L275P, and G276D (**Supplementary Figure 3**).

In CP ORF (coat protein), the nucleotide and amino acid sequences were 100% identical among two isolates obtained in this study (20SM3 and 19RP1) as well as with BMV_OH isolate (**Table 4**). The nucleotide sequences were 99.8% and 100% identical with 20SM3 and 19RP1 to BMV_OH2 and BMV_Estonia. However, the nucleotide sequences were about 96.8% and 97.4% amino acid identity between BMV_CZ isolate compared with 20SM3 and 19RP1. BMV_CZ had 95.8% to 96.8% nucleotide sequence and 97.4% to 97.9% amino acid sequence identity with other isolates analyzed in this study. The Czech isolate has five amino acid substitutions including R22P, T24A, A25V, R26K, and A124V (**Supplementary Figure 4**). BMV_OK (R26T and L35F) and BMV_UK (R26G and V100I) have two amino acid substitutions and BMV_M1 (R23W) and BMV_M2 (A25T) have one amino acid substitution (**Supplementary Figure 4**).

### Phylogenetic analysis

**Figure 4:**
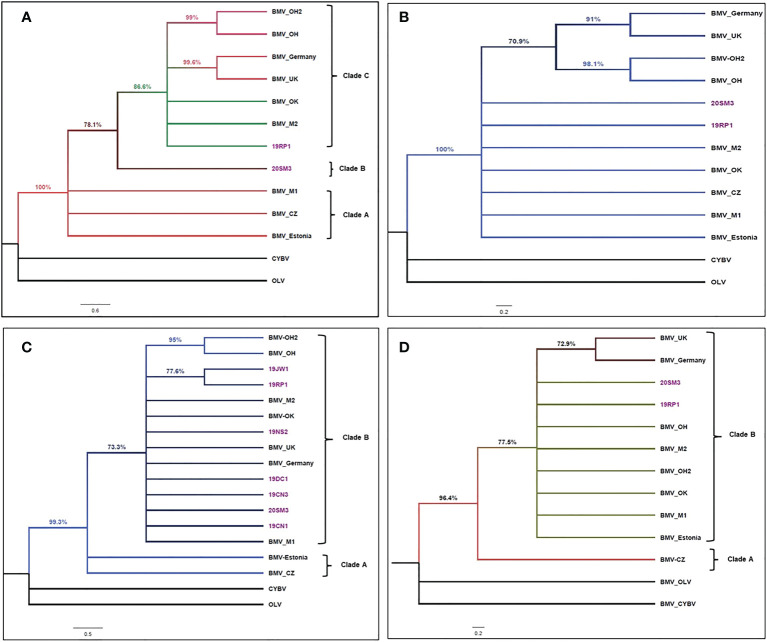
Cladogram of brome mosaic virus isolates. **(A)**. Cladogram of brome mosaic virus (BMV) isolates based on the coding sequence alignment of RNA1a sequenced in this study (highlighted in purple text) and selected strains retrieved from GenBank. The phylogenetic tree was made using the maximum likelihood analysis with a TN93 + G substitution model conducted in MEGA X (Kumar et al., 2018). The tree with the highest log likelihood (-11086.86) is shown. The percentage of replicate trees in which the associated taxa clustered together based on 1000 bootstrap replicates is presented. The posterior probability of 70% was the cutoff value and branches not supported were collapsed. Cassia yellow blotch virus (CYBV) and Olive latent virus2 (OLV) were used as outgroups in the analysis. Brackets on the right side indicate the taxa clustered in BMV clades A to C **(B)**. Cladogram of brome mosaic virus (BMV) isolates based on the coding sequence alignment of RNA2a sequenced in this study (highlighted in purple text) and selected strains retrieved from GenBank. The phylogenetic tree was made using the maximum likelihood analysis with a TN93 + G substitution model conducted in MEGA X (Kumar et al., 2018). The tree with the highest log likelihood (-9746.52) is shown. The percentage of replicate trees in which the associated taxa clustered together based on 1000 bootstrap replicates is presented. The posterior probability of 70% was the cutoff value and branches not supported were collapsed. Cassia yellow blotch virus (CYBV) and Olive latent virus 2 (OLV) were used as outgroups in the analysis. **(C)**. Cladogram of brome mosaic virus (BMV) isolates based on the coding sequence alignment of RNA3a (movement protein) sequenced in this study (highlighted in purple text) and selected strains retrieved from GenBank. The phylogenetic tree was made using the maximum likelihood analysis with a K2 + I substitution model conducted in MEGA X (Kumar et al., 2018). The tree with the highest log likelihood (-4171.53) is shown. The percentage of replicate trees in which the associated taxa clustered together based on 1000 bootstrap replicates is presented. The posterior probability of 70% was the cutoff value and branches not supported were collapsed. Cassia yellow blotch virus (CYBV) and Olive latent virus 2 (OLV) were used as outgroups in the analysis. Brackets on the right side indicate the taxa clustered in BMV clades A and B **(D)**. Cladogram of brome mosaic virus (BMV) isolates based on the coding sequence alignment of RNA4 (coat protein) sequenced in this study (highlighted in purple text) and selected strains retrieved from GenBank. The phylogenetic tree was made using the maximum likelihood analysis with a K2 + I substitution model conducted in MEGA X (Kumar et al., 2018). The tree with the highest log likelihood (-2359.86) is shown. The percentage of replicate trees in which the associated taxa clustered together based on 1000 bootstrap replicates is presented. The posterior probability of 70% was the cutoff value and branches not supported were collapsed. Cassia yellow blotch virus (CYBV) and Olive latent virus 2 (OLV) were used as outgroups in the analysis. Brackets on the right side indicate the taxa clustered in BMV clades A and B.

The sequence of coding regions of RNA1a, RNA2a, and RNA4 of 11 BMV isolates, two isolates from this study, and nine isolates obtained from GenBank (**Supplementary Table 3**) were used to construct the three cladograms separately (**Figures 4A, B, D**). We obtained complete sequences of RNA3a of seven isolates from this study and the cladograms constructed of a total of 16 BMV isolates (**Figure 4C**), nine isolates obtained from GenBank (**Supplementary Table 1**). Cassia yellow blotch virus (CYBV) and Olive latent virus 2 (OLV) were used as outgroups in the analysis.

The eleven BMV isolates used in this study were grouped into separate clades depending upon the RNA genome. The coding sequence of RNA1a of 11 BMV isolates consists of three clades (**Figure 4A**). Clade A was represented by three isolates: Czech, Estonian, and a Wisconsin isolate from the US. Clade B was represented by a single isolate 20SM3 from Smith County, KS. Clade C included isolates from the US, UK, and Germany. In clade C, two isolates from Ohio and isolates from UK and Germany form separate sister taxa groups (**Figure 4A**).

Based on the coding sequence of RNA2 of 11 BMV isolates, the BMV RNA2a topology grouped into a single clade (**Figure 4B**). However, similar two isolates from Ohio and isolates from UK and Germany form a sub-clade with separate sister taxa groups (**Figure 4B**).

The topology constructed using the coding sequence of the movement protein of BMV consisted of two clades (**Figure 4C**). Clade A included the isolates from the Czech Republic and Estonia. Clade B polytomies included isolates from the UK, Germany, and other US isolates. The isolates collected from Jewell (19JW1) and Republic (19RP1) Kansas counties form a sister taxa group (**Figure 4C**). The other three isolates collected from Smith (20SM3), Ness (19NS2), Cheyenne (19CN1 and 19CN3), and Decatur (19DC1) counties form the polytomy in clade B.

Similarly, the cladogram constructed using coding sequences of RNA4 (Coat protein) also consists of two clades (**Figure 4D**). Clade A was represented a single BMV Czech isolate. Clade B consisted of all US isolates and isolate from Estonia, the UK, and Germany. In Clade B, a sister taxa group was formed by isolates from Germany and UK.

### Population genetic parameters and neutrality tests

The population genetic parameters including nucleotide diversity, mutation, and mutation rate per segregating site of BMV US isolates were calculated using DnaSP 5.10 (**Table 5**). RNA2a exhibited the highest diversity (π = 0.01), while RNA1a and RNA3a showed the lowest diversity (π = 0.004). The degree of constrains for amino acid changes measured by the dN/dS for each encoded region showed that RNA3a was the least tolerant region with the order of tolerance RNA1a > RNA2a > RNA3b > RNA4 compared to RNA3a and RNA4.

**Table 5:** Population genetics parameters for encoded region of selected United States brome mosaic virus isolates calculated using DnaSP (Librado and Rozas, 2009) and MEGA X (Kumar et al., 2018).

Genomic region	Number of isolates	S^*^	η^†^	π^‡^	θw^#^	dS^§^	dN^ǂ^	dN/Ds (ω)^ψ^
RNA1a	5	30	30	0.0048 ± 0.001	0.0049 ± 0.003	0.013 ± 0.003	0.001 ± 0.0004	0.077
RNA2a	7	79	79	0.01 ± 0.003	0.013 ± 0.006	0.027 ± 0.003	0.002 ± 0.001	0.074
RNA3a (MP)	12	15	15	0.004 ± 0.0008	0.005 ± 0.002	0.007 ± 0.003	0.003 ± 0.001	0.428
RNA3a (CP)	7	12	12	0.006 ± 0.002	0.008 ± 0.004	0.014 ± 0.005	0.003 ± 0.001	0.214

*Total number of segregating sites.

†Total number of mutations.

‡Overall mean diversity with the standard deviation calculated by DnaSP.

#Estimated mutation rate using segregation sites.

§Number of synonymous substitutions per site from the overall mean of sequence pairs.

ǂNumber of non-synonymous substitutions per site from the overall mean of sequence pairs.

ΨRatio of dN/Ds used to determine the selective pressure for coding regions.

The dN/dS ratio of the number of nonsynonymous substitutions to the number of synonymous substitutions for all proteins coding genes was < 1 for US isolates of BMV (**Table 3**). The selection pressure was measured from the three different algorithms (FEL, FUBAR, and SLAC) and the purifying or negative selection was supported by all three methods. No positive selection pressure was significantly reported at least by two methods (data not shown).

## Discussion

Detection of wheat viruses is complicated due to the high probability of co-infection multiple viruses. Diagnostic laboratories in the Great Plains region primarily use immunological methods to confirm, which are detected singly. Nanopore technology has been previously used to successfully identify multiple wheat-infecting viruses co-infected in a single sample (Fellers et al., 2019). Accurate diagnosis of viral infections affects the management of wheat viruses through host resistance. Accurate diagnosis of viral infections affects the management of wheat viruses through host resistance. As one of the main factors that affect the durability of resistance is the dynamics of genetic variability of a pathogen (García-Arenal and McDonald, 2003). In this study, we reported the details of BMV co-infection with other wheat viruses in Kansas that were not previously identified and compared genetic variability and evolutionary characteristics with other BMV isolates obtained from this study and retrieved from the GenBank. The results of the genetic characterization of BMV isolates we recently identified in Kansas wheat fields provide information on the further study of wheat virus evolution, designing appropriate diagnostic tools, and developing durable viral disease management strategies through the breeding program.

Our results identifying BMV from 29 different counties of Kansas suggested that BMV has the potential to cause significant economic losses in Kansas wheat production. Previous studies showed that BMV reduced the wheat kernel weight and number of kernels per spike (Tošič, 1971; Pocsai et al., 1991) and total grain yield up to 61% at early stage inoculation in Ohio wheat fields (Hodge et al., 2019). Our finding of this virus coinfected with common yield-reducing wheat viruses in Kansas demands future studies to examine the bi-, tri-, quadri, or multipartite interaction of these viruses in wheat and their impact on production. A recent study reported the quadripartite infection of wheat by BMV, WSMV, TriMV, and Barley stripe mosaic virus (BSMV) resulted in severe disease synergism with the death of most infected plants (Tatineni et al., 2022). The authors also reported the titer of the viruses depends upon the types of multipartite infection. Therefore, future research should endeavor to measure the impact of multipartite infection on Kansas-adapted wheat cultivars to estimate the differential synergistic impact of viruses on Kansas wheat production.

Sequence alignment analysis of the coding regions of BMV RNAs showed that they were closely related to each other as they shared a high nucleotide sequence identity (>95%). However, US isolates showed lower similarity to Czech and Estonian isolates. Similar results were also reported by Jeżewska et al., 2019 comparing BMV isolates from Poland with BMV isolates from other European and US isolates. Gadiou and Kundu, 2013; Jeżewska et al., 2019 reported the most divergence in coat protein RNA4 region. Our results also showed higher similarities in both nucleotide and amino acid sequence of RNA3a movement protein, and US isolates had the least nucleotide sequence identity with Czech isolate in coat protein region (96.5%).

The two conserved domains of RNA1a are RNA capping enzyme domain (L52, H80, D106, and R136) helicase-like domains (K691, D755, and G781) and a polymerase-related domain of RNA2a (451 to 484) required for BMV RNA replication and mutation in or near these domains abolishes or decrease BMV RNA synthesis (Kroner et al., 1989; Koonin et al., 1991; Ahola et al., 2000). Amino acid sequence alignments of the BMV isolates in this study also maintained the integrity of the conserved domains of RNA1a and RNA2a (**Supplementary Figures 1**, **2**).

The amino acid substitutions were variable among isolates, and past studies showed that host adoption played an important role. De Jong and Ahlquist, 1995 described viral RNA accumulation in systemic infection closely associated with interaction with virus-host. They reported that both BMV_M1 and M2 strains systemically infected the monocot host barley but the dicot host cowpea was infected systemically only by BMV_M2. RNA3a movement protein and C-terminus of coat protein control the BMV movement from cell to cell and systemic movement (De Jong et al., 1995; Okinaka et al., 2001) and movement protein also played a role in host specificity (De Jong and Ahlquist, 1992; Mise et al., 1993; Mise and Ahlquist, 1995). De Jong et al., 1995 showed that quadruple substitution in BMV_M2 movement protein (E59Q, S81P, S297G, and T229S) were required to infect cowpea dicot host systemically. None of these substitutions were found in BMV_M1, the monocot-adapted isolate. Our results of BMV isolates obtained from the wheat host in this study had only one amino acid (S81P) change similar to BMV_M2 (**Supplementary Figure 3**). However, BMV_OH, BMV_OH2, and BMV_OK shared only two amino acid substitutions with BMV_M2 (S81P and T299S). Hodge et al., 2019 reported that cowpea and soybean were systematically infected by BMV_OH, which means only two amino acid substitution in MP were sufficient to infect dicot hosts. The isolates obtained in this study had only one amino acid substitution out of four essential substitutions. If these isolates can infect the dicot host with one amino acid substitution, that threatens the crop production of dicot and monocot crops due to host expansion. Therefore, future studies to determine the host ranges of Kanas BMV isolates, or survey of corn, soybean, and fescue from Kansas for detection of BMV will help to develop the management strategies of crop rotation or management of the BMV reservoir or alternative hosts.

Sacher and Ahlquist, 1989 reported that the deletion of the first 25 amino acids of BMV coat protein failed on the packaging of RNA and systemic infection. BMV isolates analyzed in this study have no change in the first 23 N-terminal amino acids in coat protein showing the conserved N-terminal region required for packaging and systemic infection. Additionally, Rao and Grantham, 1995 revealed that amino-terminal residues of 1 to 7 are required for chlorotic local lesions, and systemic infection in *Chenopodium quinoa* however did not affect barley plant infections. Therefore 1-7 N-terminal residues play important role in virus-host interactions. Deletion first 11, 14, and 18 N-terminal amino acids, especially arginine-rich motif, played a role in modulating symptom expression and movement in dicot and monocot hosts (Rao and Grantham, 1996). Okinaka et al., 2001 investigated the 19 alanine-scanning mutant, the results indicated that the C-terminal region (mainly from 178 to 187 residues) played an essential role in virus encapsidation and movement as alanine mutant on this region failed to produce virion and cell to cell movement. Yi et al., 2009 showed that three residues (D139, R142, and D 148) in the C-terminal of CP required for the BMV RNA accumulation as a mutation on these residues impact CP-associated activities. We also found no variations in the first 23 N-terminal and last 64 C-terminal amino acid residues in CP of BMV isolates analyzed in this study (**Supplementary Table 4**).

Phylogenetic relations showed modest variation among isolates with slightly different clustering based on nucleotide sequences of RNA genomes (**Figures 4A–D**) and indicated different evolutionary constraints in the different coding regions of RNAs. As only limited complete genomes of BMV are available in the GenBank, the cladograms indicated no clear grouping of isolates by geographical areas. The isolates from the Czech Republic and Estonia, UK and German, and two isolates from Ohio were most closely related to each other as they formed sister taxa in all four trees. Also, the BMV_M1 (US isolates) clustered with isolates from Estonia and the Czech Republic and 20SM3 singly formed clade B on cladogram constructed based on ORF 1a. These results suggested that although, 20SM3 was 100% similar in protein identity with 19RP1 it was sufficiently different in the nucleotide sequence, suggesting synonymous substitutions. Jeżewska et al., 2019 reported a similar clustering of the US isolates, Czech and Estonian isolates based on the CP region that we reported in this study. The slight variability among BMV isolates in different coding regions might be associated with interaction with the specific hosts and the genetic requirements to perform successful cell to cell and systemic movement (De Jong and Ahlquist, 1995; De Jong et al., 1995).

The selection pressure in protein-coding genes was calculated by nonsynonymous to synonymous substitution (dNs/Ds) ratio. The values of the dNs/Ds used to identify protein sites that experience neutral selection (dNs/Ds ≈ 1), negative or purifying selection (dNs/Ds >1), and experience positive or adaptive/diversifying selection (dNs/Ds >1) (Yang et al., 2000; Kosakovsky Pond and Frost, 2005). The ratio of the nonsynonymous and synonymous positions (dNs/Ds) estimation of all ORF of BMV genome was below one that indicates the negative or purifying selection. This result is similar to the common negative, or purifying selection of other plant RNA viruses (García-Arenal et al., 2001) as genetic stability is common. Future analysis of many BMV isolates would be needed for more accurate assessment of population parameters. The high genetic stability found for all proteins of BMV could be attributed to negative or purifying selection to maintain the functional integrity of the viral genome as described by other RNA viruses (Moreno et al., 2004). The low genetic diversity and purifying selection as common selection pressure was also reported for populations of other RNA viruses including WSMV, TriMV, cucumber mosaic virus, and Citrus psorosis virus (Stenger et al., 2002; Martín et al., 2006; Nouri et al., 2014; Redila et al., 2021). As plant RNA viruses have a short genome, each amino acid sequence contributed to encode protein. However, there is some room for the variation in RNA viruses through mutation and recombination (Drake and Holland, 1999; García-Arenal et al., 2001). The linear model of replication, preventing mutational meltdown, and genetic bottlenecks are the other reasons for low variability or neutral and purifying selection in RNA viruses (Stent, 1963; French and Stenger, 2003).

Overall, the present study characterized the newly identified BMV isolates from Kansas wheat fields. Though focused on wheat infecting wheat viruses and those with poly-A tails, this study provides meaningful evidence that BMV is commonly present in Kansas wheat. This is an indicator that in future work, a broader analysis may be warranted to detect unique viral sequences not commonly tested for in diagnostic lab samples to examine the viral population contained within a single wheat plant. This study showed the significance of nanopore sequencing in detection, diagnosis, and molecular characterization based on the whole genome sequence of undetected plant pathogens. This long-read technology has been successfully used in complete genome sequencing of plant pathogens, but also the diagnosis of unknown pathogens (Hamim et al., 2022; Sun et al., 2022). Nanopore sequencing technology uses single-molecule sequencing technology and can provide cost-effective, rapid results when compared to alternate systems, making it a good choice for diagnostics (Villamor et al., 2019). Furthermore, ONT allows for greater resolution of mixed viral infections (Liefting et al., 2021). Information of genetic variation, phylogenetic relationship with the other US and non-US isolates, and evolutionary mechanism employed by four different RNA genomes of BMV would support the sustainable management of wheat viruses through genetic resistance.

## Data availability statement

The datasets presented in this study can be found in online repositories. The names of the repository/repositories and accession number(s) can be found below: https://www.ncbi.nlm.nih.gov/, SRA PRJNA915982.

## Author contributions

Conceptualization: JR, MB, and NR. Design and methodology: NR, MB, and JR. Experiment execution and analysis: NR. The first original draft preparation: NR. Writing, rewriting and editing: NR, MB, JR, and JF. Supervision: JR. Bioinformatics: JF and NR. All authors read and approved the final manuscript. All authors contributed to the article and approved the submitted version.
