# Supplementary material for: Brome mosaic virus detected in Kansas wheat co-infected with other common wheat viruses
Source: Front Plant Sci. 2023 Mar 3;14:1096249. doi: 10.3389/fpls.2023.1096249 (PMC10022736; doi:10.3389/fpls.2023.1096249)
Supplement: Supplementary file 1 [file Image_1.pdf]

Supplementary Figure 1. Amino acid sequence alignment diagram for RNA1a of brome mosaic virus. The alignment was obtained by using muscle alignment in Mega X. Identical amino acids in all isolates are indicated by dots. The substitution of amino acid in some isolates appears as a letter

```
                      10        20        30        40        50
20SM3        MSSSIDLLKLIAEKGAASQSAQDIVDNQVAQQLSAQIEYAKRSKKINVRN
19RP1        ..................................................
BMV-OH2      ..................................................
BMV-OH       ..................................................
BMV-M1       ................D.................................
BMV-M2       ..................................................
BMV-OK       ..................................................
BMV-Germany  ................V....L............................
BMV-UK       ................V....L............................
BMV-Estonia  ................E.................................
BMV-CZ       ................D.................................
```

```
                      60        70        80        90       100
20SM3        KLSIEEADAFRDRYGGAFDLNLTQQYHAPHSLAGALRVAEHYDCLDSFPP
19RP1        ..................................................
BMV-OH2      ..................................................
BMV-OH       ..................................................
BMV-M1       ..................................................
BMV-M2       ..................................................
BMV-OK       ..................................................
BMV-Germany  ..................................................
BMV-UK       ..................................................
BMV-Estonia  ..................................................
BMV-CZ       .........................................F........
```

```
                     110       120       130       140       150
20SM3        EDPVIDFGGSWWHHFSRRDKRVHSCCPVLGVRDAARHEERMCRMRKILQE
19RP1        ..................................................
BMV-OH2      ..................................................
BMV-OH       ..................................................
BMV-M1       ..................................................
BMV-M2       ..................................................
BMV-OK       ..................................................
BMV-Germany  ..................................................
BMV-UK       ..................................................
BMV-Estonia  ..................................................
BMV-CZ       ..................................................
```

```
                     160       170       180       190       200
20SM3        SDDFDEVPNFCLNRAQDCDVQADWAICIHGGYDMGFQGLCDAMHSHGVRV
19RP1        ..................................................
BMV-OH2      ..................................................
BMV-OH       ..................................................
BMV-M1       ..................................................
BMV-M2       ..................................................
BMV-OK       ..................................................
BMV-Germany  ..................................................
BMV-UK       ..................................................
BMV-Estonia  ..................................................
BMV-CZ       ..................................................
```

```
                     210       220       230       240       250
20SM3        LRGTVMFDGAMLFDREGFLPLLKCHWQRDGSGADEVIKFDFENESTLSYI
19RP1        ..................................................
BMV-OH2      ..................................................
BMV-OH       ..................................................
BMV-M1       ..................................................
```

```
BMV-M2       ..................................................
BMV-OK       ..................................................
BMV-Germany  ..................................................
BMV-UK       ..................................................
BMV-Estonia  ................................T.................
BMV-CZ       ................................T.................

                      260       270       280       290       300
             ....|....|....|....|....|....|....|....|....|....|
20SM3        HGWQDLGSFFTESVHCIDGTTYLLEREMLKCNIMTYKIIATNLRCPRETL
19RP1        ..................................................
BMV-OH2      ...R..............................................
BMV-OH       ...R..............................................
BMV-M1       ..................................................
BMV-M2       ..................................................
BMV-OK       ..................................................
BMV-Germany  ..................................................
BMV-UK       ..................................................
BMV-Estonia  ..................................................
BMV-CZ       ..................................................

                      310       320       330       340       350
             ....|....|....|....|....|....|....|....|....|....|
20SM3        RHCVWFEDISKYVGVSIPEDWSLNRWKCVRVAKTTVREVEEIAFRCFKEN
19RP1        ..................................................
BMV-OH2      ..................................................
BMV-OH       ..................................................
BMV-M1       .................................................S
BMV-M2       ..................................................
BMV-OK       ..................................................
BMV-Germany  ..................................................
BMV-UK       ..................................................
BMV-Estonia  ..................................................
BMV-CZ       ..................................................

                      360       370       380       390       400
             ....|....|....|....|....|....|....|....|....|....|
20SM3        KEWTENMKAVASILSAKSSTVIINGQAIMAGERLDIEDYHLVAFALTLNL
19RP1        ..................................................
BMV-OH2      ..................................................
BMV-OH       ..................................................
BMV-M1       ..................................................
BMV-M2       ..................................................
BMV-OK       ..................................................
BMV-Germany  ..................................................
BMV-UK       ..................................................
BMV-Estonia  ..................................................
BMV-CZ       ..................................................

                      410       420       430       440       450
             ....|....|....|....|....|....|....|....|....|....|
20SM3        YQKYEKLTALRDGMEWKGWCHHFKTRFWWGGDSSRAKVGWLRTLASRFPL
19RP1        ..................................................
BMV-OH2      ..................................................
BMV-OH       ..................................................
BMV-M1       ..................................................
BMV-M2       ........V.........................................
BMV-OK       ..................................................
BMV-Germany  ..................................................
BMV-UK       ..................................................
BMV-Estonia  ..................................................
BMV-CZ       ..................................................
```

```
                    460       470       480       490       500
20SM3        LRLDSYADSFKFLTRLSNVEEFEQDSVPISRLRTFWTEEDLFDRLEHEVQ
19RP1        ..................................................
BMV-OH2      ..................................................
BMV-OH       ..................................................
BMV-M1       ..................................................
BMV-M2       ..................................................
BMV-OK       ..................................................
BMV-Germany  ..................................................
BMV-UK       ..................................................
BMV-Estonia  ..................................................
BMV-CZ       ..................................................

                    510       520       530       540       550
20SM3        TAKTKRSKKKAKVPPAAEIPQEEFHDAPESSSPESVSDDVKPVTDVVPDA
19RP1        ..................................................
BMV-OH2      ...........I................................A.....
BMV-OH       ...........T................................A.....
BMV-M1       ..................................................
BMV-M2       ..................................................
BMV-OK       ...........I......................................
BMV-Germany  ..................................................
BMV-UK       ..................................................
BMV-Estonia  ..........................................E.....G.
BMV-CZ       ..................M.............................G.

                    560       570       580       590       600
20SM3        EVSVEVPTDPRGISRHGAMKEFVRYCKRLHNNSESNLRHLWDISGGRGSE
19RP1        ..................................................
BMV-OH2      ..................................................
BMV-OH       .......S..........................................
BMV-M1       ..................................................
BMV-M2       ..................................................
BMV-OK       ..................................................
BMV-Germany  ..................................................
BMV-UK       ..................................................
BMV-Estonia  ..................................................
BMV-CZ       ....A.............................................

                    610       620       630       640       650
20SM3        IANKSIFETYHRIDDMVNVHLANGNWLYPKKYDYTVGYNEHGLGPKHADE
19RP1        ..................................................
BMV-OH2      ..................................................
BMV-OH       ..................................................
BMV-M1       ..................................................
BMV-M2       ..................................................
BMV-OK       ..................................................
BMV-Germany  ..................................................
BMV-UK       ..................................................
BMV-Estonia  ..................................................
BMV-CZ       .........................................S........

                    660       670       680       690       700
20SM3        TYIVDKTCACSNLRDIAEASAKVSVPTCDISMVDGVAGCGKTTAIKDAFR
19RP1        ..................................................
BMV-OH2      ..................................................
BMV-OH       ..................................................
BMV-M1       ..................................................
```

```
BMV-M2        ..................................................
BMV-OK        ..................................................
BMV-Germany   ..................................................
BMV-UK        ..................................................
BMV-Estonia   ..................................................
BMV-CZ        ..................................................

                       710       720       730       740       750
20SM3         MGEDLIVTANRKSAEDVRMALFPDTFNSKVALDVVRTADSAIMHGVPSCH
19RP1         ..................................................
BMV-OH2       ..................................................
BMV-OH        ..................................................
BMV-M1        .........................Y........................
BMV-M2        ..................................................
BMV-OK        ..................................................
BMV-Germany   ..................................................
BMV-UK        ..................................................
BMV-Estonia   ..................................................
BMV-CZ        .................................................Y

                       760       770       780       790       800
20SM3         RLLVDEAGLLHYGQLLVVAALSKCSQVLAFGDTEQISFKSRDAGFKLLHG
19RP1         ..................................................
BMV-OH2       ..................................................
BMV-OH        ..................................................
BMV-M1        ..................................................
BMV-M2        ..................................................
BMV-OK        ..................................................
BMV-Germany   ..................................................
BMV-UK        ..................................................
BMV-Estonia   ..................................................
BMV-CZ        ..................................................

                       810       820       830       840       850
20SM3         NLKYDRRDVVHKTYRCPQDVIAAVNLLKRKCGNRDTKYQSWTSESKVSRS
19RP1         ..................................................
BMV-OH2       ..................................................
BMV-OH        ..................................................
BMV-M1        ..Q...............................................
BMV-M2        .......................I..........................
BMV-OK        ..................................................
BMV-Germany   ..................................................
BMV-UK        ..................................................
BMV-Estonia   ..Q...............................................
BMV-CZ        ..Q...............................................

                       860       870       880       890       900
20SM3         LTKRRITSVLQVTIDPNRTYLTMTQADKAALQTRAKDFPVSKDWIDGHIK
19RP1         ..................................................
BMV-OH2       ..................................................
BMV-OH        ..................................................
BMV-M1        ........G.........................................
BMV-M2        ......A...........................................
BMV-OK        ..................................................
BMV-Germany   .A................................................
BMV-UK        .A................................................
BMV-Estonia   ..................................................
BMV-CZ        ........G.........................................
```

```
                     910       920       930       940       950
             ....|....|....|....|....|....|....|....|....|....|
20SM3        TVHEAQGISVDNVTLVRLKSTKCDLFKHEEYCLVALTRHKKSFEYCFNGE
19RP1        ..................................................
BMV-OH2      ..................................................
BMV-OH       ..................................................
BMV-M1       ..................................................
BMV-M2       ..................................................
BMV-OK       .............A....................................
BMV-Germany  ..................................................
BMV-UK       ..................................................
BMV-Estonia  ..................................................
BMV-CZ       ..................................................

                     960
             ....|....|.
20SM3        LAGDLIFNCVK
19RP1        ...........
BMV-OH2      ...........
BMV-OH       ...........
BMV-M1       ...........
BMV-M2       ...........
BMV-OK       ...........
BMV-Germany  ...........
BMV-UK       ...........
BMV-Estonia  ...........
BMV-CZ       ...........
```
