# Supplementary material for: Brome mosaic virus detected in Kansas wheat co-infected with other common wheat viruses
Source: Front Plant Sci. 2023 Mar 3;14:1096249. doi: 10.3389/fpls.2023.1096249 (PMC10022736; doi:10.3389/fpls.2023.1096249)
Supplement: Supplementary file 2 [file Image_2.pdf]

Supplementary Figure 2. Amino acid sequence alignment diagram for RNA2b of brome mosaic virus. The alignment was obtained by using muscle alignment in MEGA X. Identical amino acids in all isolates are indicated by dots. The substitution of amino acid in some isolates appears as a letter

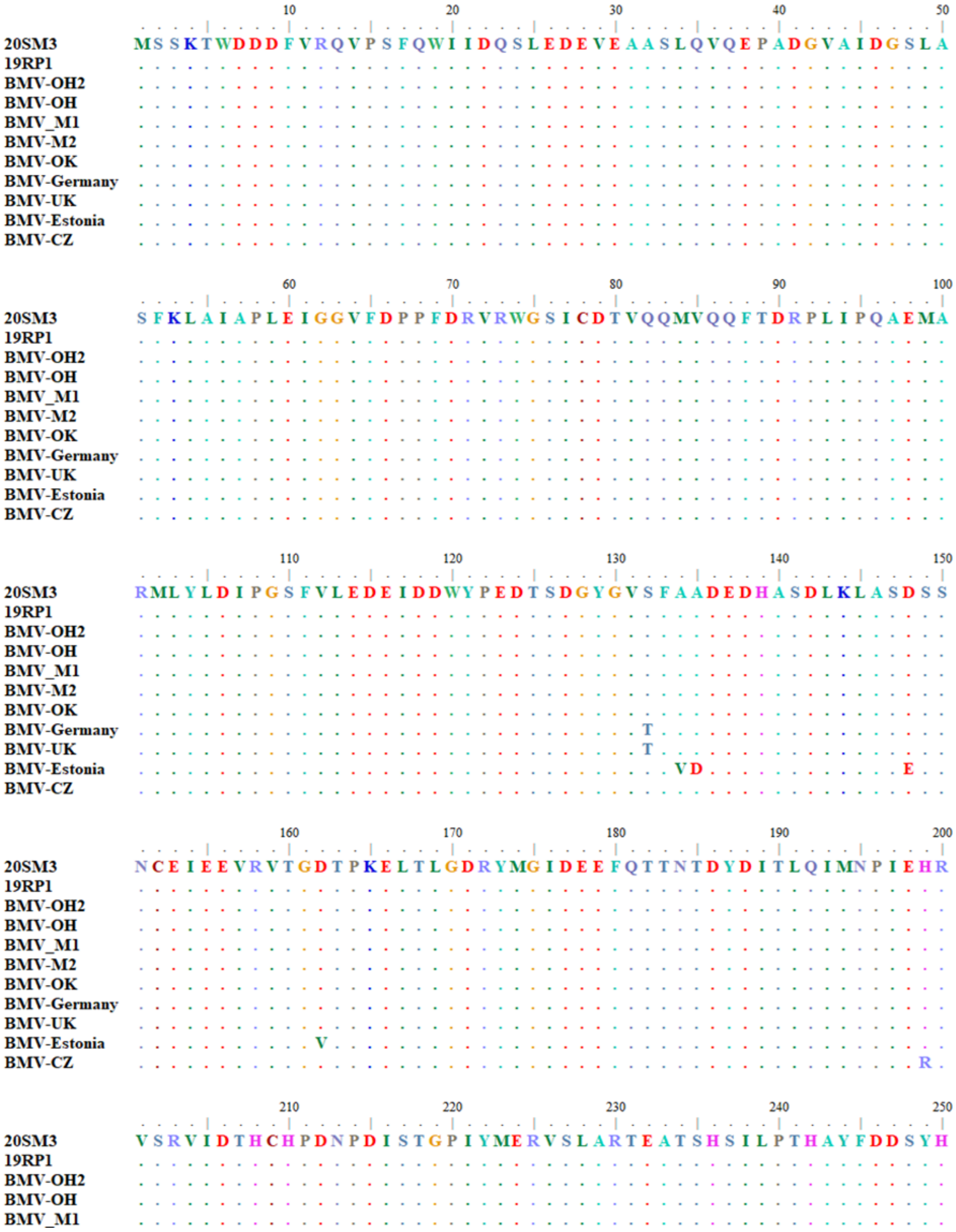

```
BMV-M2       ..................................................
BMV-OK       ..................................................
BMV-Germany  ..................................................
BMV-UK       ..................................................
BMV-Estonia  ......................L...........................
BMV-CZ       ..................................................

                      260       270       280       290       300
             ....|....|....|....|....|....|....|....|....|....|
20SM3        QALVENGDYSMDFDRIRLKQSDVDWYRDPDKYFQPKMNIGSAQRRVGTQK
19RP1        ..................................................
BMV-OH2      ..................................................
BMV-OH       ..................................................
BMV_M1       ..................................................
BMV-M2       ..................................................
BMV-OK       ..................................................
BMV-Germany  ..................................................
BMV-UK       ..........................K.......................
BMV-Estonia  ..................................................
BMV-CZ       ..........................K...R...................

                      310       320       330       340       350
             ....|....|....|....|....|....|....|....|....|....|
20SM3        EVLTALKKRNADVPEMGDAINMKETAKAIAKRFRSTFLNVDGEDCLRASM
19RP1        ..................................................
BMV-OH2      ..................................................
BMV-OH       ..................................................
BMV_M1       .......................D..........................
BMV-M2       ..................................................
BMV-OK       ..................................................
BMV-Germany  ..................................................
BMV-UK       ..................................................
BMV-Estonia  .......................D......E...................
BMV-CZ       .......................D..........................

                      360       370       380       390       400
             ....|....|....|....|....|....|....|....|....|....|
20SM3        DVMTKCLEYHKKWGKHMDLQGVNVAAETDLRRYQHMLKSDVKPVVTDTLH
19RP1        ..................................................
BMV-OH2      ..................................................
BMV-OH       ..................................................
BMV_M1       ..............................C...................
BMV-M2       ..................................................
BMV-OK       ..................................................
BMV-Germany  ..................................................
BMV-UK       ..................................................
BMV-Estonia  ..............................C...................
BMV-CZ       ..............................C...................

                      410       420       430       440       450
             ....|....|....|....|....|....|....|....|....|....|
20SM3        LERAVAATITFHSKGVTSNFSPFFTACFEKLSLALKSRFIVPIGKISSLE
19RP1        ..................................................
BMV-OH2      ..................................................
BMV-OH       ..................................................
BMV_M1       ..................................................
BMV-M2       ..................................................
BMV-OK       ..................................................
BMV-Germany  ..................................................
BMV-UK       ..................................................
BMV-Estonia  ..................................................
BMV-CZ       ..................................................
```

```
                  460       470       480       490       500
20SM3        LKNVRLNNRYFLEADLSKFDKSQGELHLEFQREILLALGFPAPLTNWWSD
19RP1        ..................................................
BMV-OH2      ..................................................
BMV-OH       ..................................................
BMV_M1       ..................................................
BMV-M2       ..................................................
BMV-OK       ..................................................
BMV-Germany  ..................................................
BMV-UK       ..................................................
BMV-Estonia  ..................................................
BMV-CZ       ..................................................

                  510       520       530       540       550
20SM3        FHRDSYLSDPHAKVGMSVSFQRRTGDAFTYFGNTLVTMAMIAYASDLSDC
19RP1        ..................................................
BMV-OH2      ..................................................
BMV-OH       ..................................................
BMV_M1       ..................................................
BMV-M2       ..................................................
BMV-OK       ..................................................
BMV-Germany  ..................................................
BMV-UK       ..................................................
BMV-Estonia  ..................................................
BMV-CZ       ..................................................

                  560       570       580       590       600
20SM3        DCAIFSGDDSLIISKVKPVLDTDMFTSLFNMEIKVMDPSVPYVCSKFLVE
19RP1        ..................................................
BMV-OH2      ..................................................
BMV-OH       ..................................................
BMV_M1       ..................................................
BMV-M2       ................R.................................
BMV-OK       ..................................................
BMV-Germany  ..................................................
BMV-UK       ..................................................
BMV-Estonia  ..................................................
BMV-CZ       ..................................................

                  610       620       630       640       650
20SM3        TEMGNLVSIPDPMREIQRLAKRKILRDEQMLRAHFVSFCDRMKFINQLDE
19RP1        ..................................................
BMV-OH2      ..................................................
BMV-OH       ..................................................
BMV_M1       ........V...L.....................................
BMV-M2       ..................................................
BMV-OK       .....W....................E.......................
BMV-Germany  ..................................................
BMV-UK       ..................................................
BMV-Estonia  ............L.....................................
BMV-CZ       ............L.......R.............................

                  660       670       680       690       700
20SM3        KMITMLCHFVYLKYGKKKPWIFEEVRAALAAFSLYSENFLRFSDCYCTEG
19RP1        ..................................................
BMV-OH2      ................E.................................
BMV-OH       ................E.................................
BMV_M1       ....T...........E.................................
```

```
BMV-M2       ................E..................................
BMV-OK       ................E..................................
BMV-Germany  ................E..................................
BMV-UK       ................E..................................
BMV-Estonia  ................E.........S........................
BMV-CZ       ................E..................................

                      710       720       730       740       750
             ....|....|....|....|....|....|....|....|....|....|
20SM3        IRVYQMSDPVCKFKRTTEERKTDGDWFHNWKNPKFPGVLDKVYRTLGIYS
19RP1        ..................................................
BMV-OH2      ..................................................
BMV-OH       ..................................................
BMV_M1       .............................................I....
BMV-M2       ................M.................................
BMV-OK       ................M.................................
BMV-Germany  ..................................................
BMV-UK       ..................................................
BMV-Estonia  ..................................................
BMV-CZ       ..................................................

                      760       770       780       790       800
             ....|....|....|....|....|....|....|....|....|....|
20SM3        SDCSTKELPVKRIGRLHEALERESLKLANDRRTTQRLKKKVDDYATGRGG
19RP1        ..................................................
BMV-OH2      ..................................................
BMV-OH       ..................................................
BMV_M1       ..................................................
BMV-M2       ..................................................
BMV-OK       .........................R........................
BMV-Germany  .................................A................
BMV-UK       .................................A................
BMV-Estonia  ..................................................
BMV-CZ       ...............S..................................

                      810       820
             ....|....|....|....|..
20SM3        LTSVDALLLKSHCETFKPSDLR
19RP1        ......................
BMV-OH2      ......................
BMV-OH       ......................
BMV_M1       ........V.............
BMV-M2       ......................
BMV-OK       ......................
BMV-Germany  ......................
BMV-UK       ......................
BMV-Estonia  ........V.............
BMV-CZ       ........V.............
```
