# Supplementary material for: Brome mosaic virus detected in Kansas wheat co-infected with other common wheat viruses
Source: Front Plant Sci. 2023 Mar 3;14:1096249. doi: 10.3389/fpls.2023.1096249 (PMC10022736; doi:10.3389/fpls.2023.1096249)
Supplement: Supplementary file 3 [file Image_3.pdf]

Supplementary Figure 3. Amino acid sequence alignment diagram for RNA3a (Movement protein) of brome mosaic virus. The alignment was obtained by using muscle alignment in Mega. Identical amino acids in all isolates are indicated by dots. The substitution of amino acid in some isolates appears as a letter.

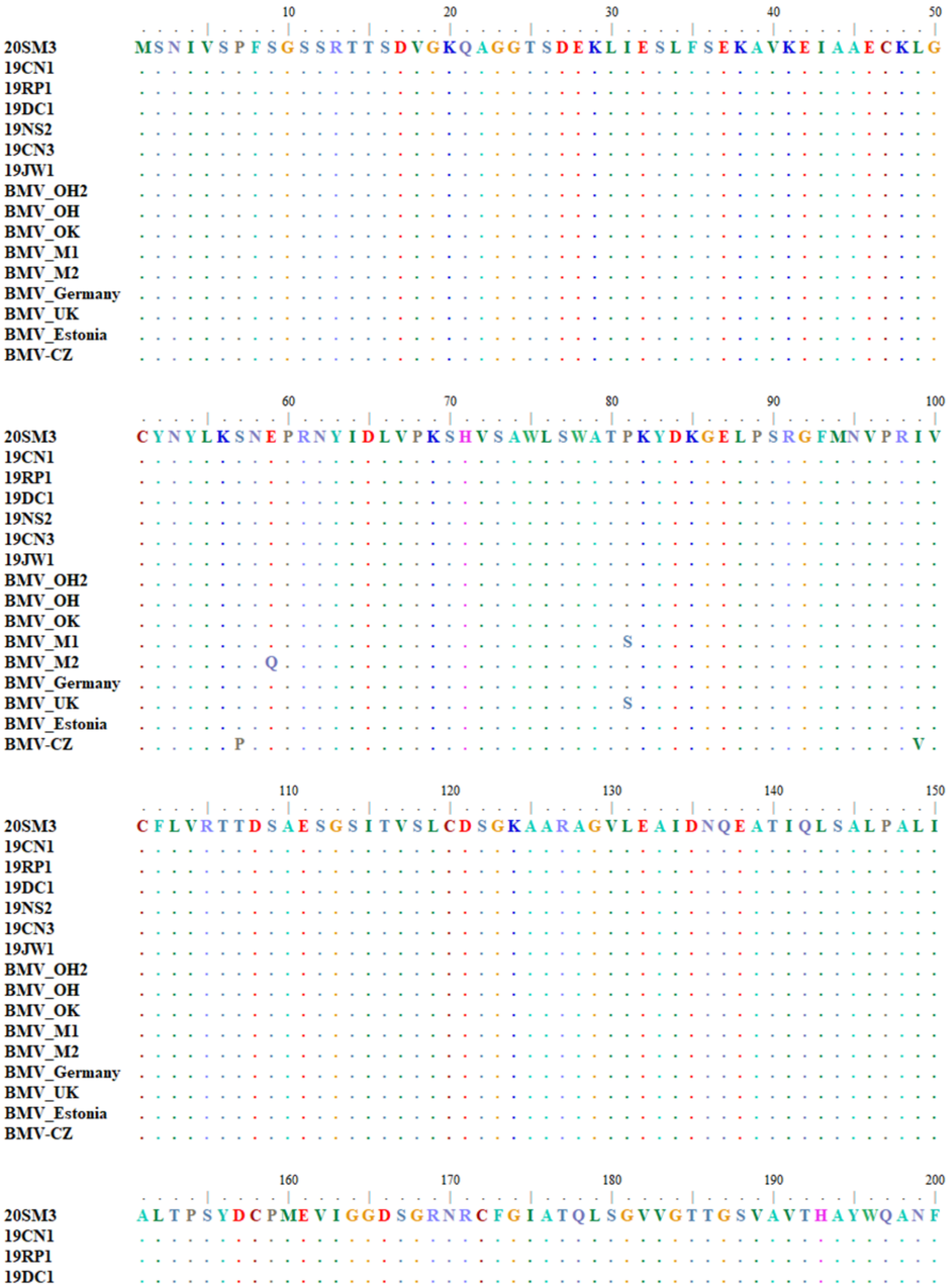

```
19NS2         ...............N..................................
19CN3         ..................................................
19JW1         ..................................................
BMV_OH2       ...........I......................................
BMV_OH        ...........I......................................
BMV_OK        ..................................................
BMV_M1        ..................................................
BMV_M2        ..................................................
BMV_Germany   ...............H..................................
BMV_UK        ...............H..................................
BMV_Estonia   ..................................................
BMV-CZ        ..................................................

                     210       220       230       240       250
20SM3         KAKPNNYKLHGPATIMVMPFDRLRQLDKKSLKNYIRGISNQSVDHGYLLG
19CN1         ..................................................
19RP1         ..................................................
19DC1         ..................................................
19NS2         ..................................................
19CN3         ..................................................
19JW1         ..................................................
BMV_OH2       ..................................................
BMV_OH        ..................................................
BMV_OK        ..................................................
BMV_M1        ..................................................
BMV_M2        ..................................................
BMV_Germany   ..................................................
BMV_UK        ..................................................
BMV_Estonia   ..................................................
BMV-CZ        ........................R.........................

                     260       270       280       290       300
20SM3         RPLQSVDQVAQEDLLVEESESPSALGRGVKDSKSVSASSVAGLPVSSPTL
19CN1         ..................................................
19RP1         ........................F.........................
19DC1         ..................................................
19NS2         ..................................................
19CN3         ..................................................
19JW1         ........................F.........................
BMV_OH2       ................................................S.
BMV_OH        ................................................S.
BMV_OK        ................................................S.
BMV_M1        ..................................................
BMV_M2        ..............................................G.S.
BMV_Germany   ..................................................
BMV_UK        ..................................................
BMV_Estonia   ..................................................
BMV-CZ        ........................PD........................

20SM3         RIK
19CN1         ...
19RP1         ...
19DC1         ...
19NS2         ...
19CN3         ...
19JW1         ...
BMV_OH2       ...
BMV_OH        ...
BMV_OK        ...
```
