# Supplementary material for: Brome mosaic virus detected in Kansas wheat co-infected with other common wheat viruses
Source: Front Plant Sci. 2023 Mar 3;14:1096249. doi: 10.3389/fpls.2023.1096249 (PMC10022736; doi:10.3389/fpls.2023.1096249)
Supplement: Supplementary file 4 [file Image_4.pdf]

**Supplementary Figure 4.** Amino acid sequence alignment diagram for coat protein of brome mosaic virus. The alignment was obtained by using muscle alignment in Mega X. Identical amino acids in all isolates are indicated by dots. The substitution of amino acid in some isolates appears as a letter

```
                      10        20        30        40        50
20SM3          MSTSGTGKMTRAQRRAAARRNRRTARVQPVIVEPLAAGQGKAIKAIAGYS
19RP1          ..................................................
BMV-OH2        ..................................................
BMV-OH         ..................................................
BMV-OK         .........................T........F...............
BMV-M1         ......................W...........................
BMV-M2         ........................T.........................
BMV_Germany    ..................................................
BMV-UK         .........................G........I...............
BMV_Estonia    ..................................................
BMV-CZ         .....................P.AVK........................

                      60        70        80        90       100
20SM3          ISKWEASSDAITAKATNAMSITLPHELSSEKNKELKVGRVLLWLGLLPSV
19RP1          ..................................................
BMV-OH2        ..................................................
BMV-OH         ..................................................
BMV-OK         ..................................................
BMV-M1         ..................................................
BMV-M2         ..................................................
BMV_Germany    ..................................................
BMV-UK         .................................................I
BMV_Estonia    ..................................................
BMV-CZ         ..................................................

                     110       120       130       140       150
20SM3          AGRIKACVAEKQAQAEAAFQVALAVADSSKEVVAAMYTDAFRGATLGDLL
19RP1          ..................................................
BMV-OH2        ..................................................
BMV-OH         ..................................................
BMV-OK         ..................................................
BMV-M1         ..................................................
BMV-M2         ..................................................
BMV_Germany    ..................................................
BMV-UK         ..................................................
BMV_Estonia    ..................................................
BMV-CZ         .......................V..........................

                     160       170       180
20SM3          NLQIYLYASEAVPAKAVVVHLEVEHVRPTFDDFFTPVYR
19RP1          .......................................
BMV-OH2        .......................................
BMV-OH         .......................................
BMV-OK         .......................................
BMV-M1         .......................................
BMV-M2         .......................................
BMV_Germany    .......................................
BMV-UK         .......................................
BMV_Estonia    .......................................
BMV-CZ         .......................................
```
