# Supplementary material for: Brome mosaic virus detected in Kansas wheat co-infected with other common wheat viruses
Source: Front Plant Sci. 2023 Mar 3;14:1096249. doi: 10.3389/fpls.2023.1096249 (PMC10022736; doi:10.3389/fpls.2023.1096249)
Supplement: Supplementary file 5 [file Table_1.docx]

Supplementary Table 1. List of sequences of cereal viruses retrieved from GenBank that were used as reference genomes to get consensus sequences

| Name of virus | Accession number |
| --- | --- |
| Ageratum yellow leaf curl betasatellite | KC305091.1 |
| Agropyron mosaic virus | NC_005903.1 |
| Barley mild mosaic virus | AJ544268.1 |
| Barley stripe mosaic virus RNA1 | NC_003469.1 |
| Barley stripe mosaic virus RNA2 | NC_003481.1 |
| Barley stripe mosaic virus RNA3 | NC_003478.1 |
| Barley yellow dwarf virus-PAS | NC_002160.2 |
| Barley yellow dwarf virus-MAV | NC_003680.1 |
| Barley yellow dwarf virus-GAV | KF523382.1 |
| Barley yellow dwarf virus-PAV | EF043235.1 |
| Barley yellow striate mosaic virus, polymerase (L) gene | FJ665628 |
| Barley yellow striate mosaic virus, glycoprotein (G) gene | KP163565.1 |
| Cereal yellow dwarf virus | EF521830.1 |
| Hordeum mosaic virus | NC_005904.1 |
| Maize streak virus | AF239960.1 |
| Oat golden stripe virus RNA1 | NC_002358.1 |
| Oat golden stripe virus RNA2 | NC_002357.1 |
| Oat necrotic mottle virus | NC_005136.1 |
| Tobacco mosaic virus Queensland | AF332868 |
| Rice black streaked dwarf virus (S1) | KC134289.1 |
| Rice black streaked dwarf virus (S2) | KC134290.1 |
| Rice black streaked dwarf virus (S3) | KC134291.1 |
| Rice black streaked dwarf virus (S4) | KC134292.1 |
| Rice black streaked dwarf virus (S5) | KC134293.1 |
| Rice black streaked dwarf virus (S6) | KC134294.1 |
| Rice black streaked dwarf virus (S7) | KC134295.1 |
| Rice black streaked dwarf virus (S8) | KC134296.1 |
| Rice black streaked dwarf virus (S9) cds_AFX68415.1_1 | KC134297.1 |
| Rice black streaked dwarf virus (S9) cds_AFX68415.1_2 | KC134297.1 |
| Rice black streaked dwarf virus (S10) | KC134298.1 |
| Soil borne wheat mosaic virus RNA1 | KT736088.1 |
| Soil borne wheat mosaic virus RNA2 | KT736089.1 |
| Wheat streak mosaic virus type strain | AF285169 |
| Wheat streak mosaic virus Hoym | HG810954.1 |
| Wheat mosaic virus KS7 RNA1 | KT988860.1 |
| Wheat mosaic virus KS7 RNA2 | KT988861.1 |
| Wheat mosaic virus KS7 RNA3A | KT988862.1 |
| Wheat mosaic virus KS7 RNA3B | KT988863.1 |
| Wheat mosaic virus KS7 RNA4 | KT988864.1 |
| Wheat mosaic virus KS7 RNA5 | KT988865.1 |
| Wheat mosaic virus KS7 RNA6 | KT988866.1 |
| Wheat mosaic virus KS7 RNA7 | KT988867.1 |
| Wheat mosaic virus KS7 RNA8 | KT988868.1 |
| Wheat dwarf virus | KJ473705.1 |
| Wheat eqlid mosaic virus | NC_009805.1 |
| Wheat rosette stunt virus | AF059602.1 |
| Wheat spindle streak mosaic virus RNA1 | NC_040508.1 |
| Wheat spindle streak mosaic virus RNA2 | NC_040507.1 |
| Wheat stripe virus RNA2 | AY312434.1 |
| Wheat stripe virus RNA3 | AY312435.1 |
| Wheat stripe virus RNA4 | AY312436.1 |
| Wheat yellow mosaic virus RNA1 | AB910332.1 |
| Wheat yellow mosaic virus RNA2 | AB910336.1 |
| Foxtail mosaic virus | EF630359.1 |
| Triticum mosaic virus KS | FJ263671.1 |
| Barley virus G | KT962089.1 |
| Barley yellow mosaic virus RNA1 | AJ132268.1 |
| Barley yellow mosaic virus RNA2 | AJ132269.1 |
| Brome mosaic virus RNA1 | NC_002026.1 |
| Brome mosaic virus RNA2 | NC_002027.1 |
| Brome mosaic virus RNA3 | NC_002028.2 |
| European wheat striate mosaic virus RNA1 | MN044342.1 |
| European wheat striate mosaic virus RNA2 | MN044343.1 |
| European wheat striate mosaic virus RNA3 | MN044344.1 |
| European wheat striate mosaic virus RNA4 | MN044345.1 |
| Chinese wheat mosaic virus RNA1 | NC_002359.1 |
| Chinese wheat mosaic virus RNA2 | NC_002356.1 |
| Johnsongrass mosaic virus | KX897165.1 |
| Maize chlorotic mottle virus | X14736.2 |
| Maize yellow dwarf virus-RMV | KC921392.1 |
| Maize yellow mosaic virus-Morogoro | MW036244.1 |
| Maize yellow striate virus | KY884303.1 |
| Oat dwarf virus | KX533459.1 |
| Panicum mosaic virus | MH885652.1 |
| Ryegrass mosaic virus | MT005828.1 |
| Sitobion miscanthi flavi-like virus | MH778148.1 |
| Soil borne cereal mosaic virus RNA1 | NC_002042.1 |
| Soil borne cereal mosaic virus RNA2 | NC_002041.1 |
| Sugarcane mosaic virus | AJ297628.1 |
| Wheat leaf yellowing-assocaited virus | KY605226.1 |
| Wheat yellow dwarf virus-GPV | NC_012931.1 |
| Wheat yellow striate virus | MG604920.1 |
| Brome streak mosaic rymovirus | Z48506.1 |
