# Supplementary material for: Brome mosaic virus detected in Kansas wheat co-infected with other common wheat viruses
Source: Front Plant Sci. 2023 Mar 3;14:1096249. doi: 10.3389/fpls.2023.1096249 (PMC10022736; doi:10.3389/fpls.2023.1096249)
Supplement: Supplementary file 6 [file Table_2.docx]

Supplementary Table 2. Sequences of brome mosaic virus retrieved from GenBank and used for comparisons.

| Sample ID | RNA genome | Origin | Accession Number |
| --- | --- | --- | --- |
| BMV_OH2 | RNA1 | OH, USA | MN241035.1 |
| BMV_OH2 | RNA2 | OH, USA | MN241036.1 |
| BMV_OH2 | RNA3 | OH, USA | MN241037.1 |
| BMV_OH | RNA1 | OH, USA | MH025765.1 |
| BMV_OH | RNA2 | OH, USA | MH025766.1 |
| BMV_OH | RNA3 | OH, USA | MH025767.1 |
| BMV_OK | RNA1 | OK, USA | DQ530423.1 |
| BMV_OK | RNA2 | OK, USA | DQ530424.1 |
| BMV_OK | RNA3 | OK, USA | DQ530425.1 |
| BMV_M1* | RNA1 | WI, USA | X02380.1 |
| BMV_ M1* | RNA2 | WI, USA | X01678.1 |
| BMV_ M1* | RNA3 | WI, USA | J02042.1 |
| BMV_M2 | RNA1 | WI, USA | AB183262.1 |
| BMV_M2 | RNA2 | WI, USA | AB183263.1 |
| BMV_M2 | RNA3 | WI, USA | AB183261.1 |
| BMV_DSMZ_PV-0194 | RNA1 | UK | MW582787.1 |
| BMV_DSMZ_PV-0194 | RNA2 | UK | MW582788.1 |
| BMV_DSMZ_PV-0194 | RNA3 | UK | MW582789.1 |
| BMV_Germany | RNA3 | Germany | MT737803.1 |
| BMV_Estonia | RNA1 | Estonia | KU726253.1 |
| BMV_Estonia | RNA2 | Estonia | KU726254.1 |
| BMV_Estonia | RNA3 | Estonia | KU726255.1 |
| BMV_CZ | RNA1 | Czech | GU584131.1 |
| BMV_CZ | RNA2 | Czech | GU584130.1 |
| BMV_CZ | RNA3 | Czech | GU584129.1 |
| CYBV** | RNA1 | - | NC_006999.2 |
| CYBV** | RNA2 | - | NC_007000.2 |
| CYBV** | RNA3 | - | NC_007001.1 |
| OLV** | RNA1 | - | X94346.1 |
| OLV** | RNA2 | - | X94347.1 |
| OLV** | RNA3 | - | X76993.1 |

*These viruses were used as the reference genomes

**These viruses were used as outgroups for the phylogenetic analysis
