# Supplementary material for: Brome mosaic virus detected in Kansas wheat co-infected with other common wheat viruses
Source: Front Plant Sci. 2023 Mar 3;14:1096249. doi: 10.3389/fpls.2023.1096249 (PMC10022736; doi:10.3389/fpls.2023.1096249)
Supplement: Supplementary file 7 [file Table_3.docx]

Supplementary Table 3. List of survey samples positive to brome mosaic virus and number of raw reads obtained from Nanopore sequencing

| **Sample ID** | **County** | **Year collected** | **Number of total reads** |
| --- | --- | --- | --- |
| 19CN1 | Cheyenne | 2019 | 771585 |
| 19CN3 | Cheyenne | 2019 | 244288 |
| 19CY4 | Clay | 2019 | 358208 |
| 19DC1 | Decatur | 2019 | 1016763 |
| 19GT | Grant | 2019 | 249860 |
| 19JW1 | Jewell | 2019 | 282607 |
| 19MT | Morton | 2019 | 295365 |
| 19NS2 | Ness | 2019 | 221786 |
| 19OB1 | Osborne | 2019 | 221669 |
| 19PL1 | Phillips | 2019 | 136534 |
| 19PN1 | Pawnee | 2019 | 609762 |
| 19PN2 | Pawnee | 2019 | 130358 |
| 19RA3 | Rawlins | 2019 | 565130 |
| 19RH1 | Rush | 2019 | 344955 |
| 19RO1 | Rooks | 2019 | 145552 |
| 19RP1 | Republic | 2019 | 608705 |
| 19RS2 | Russell | 2019 | 269145 |
| 19SV | Stevens | 2019 | 557096 |
| 19SW | Seward | 2019 | 401427 |
| 19TH2 | Thomas | 2019 | 52369 |
| 19TR1 | Trego | 2019 | 654870 |
| 20 LE1 | Lane | 2020 | 90608 |
| 20SD4 | Sheridan | 2020 | 1294849 |
| 20GL2 | Greeley | 2020 | 106591 |
| 20TR2 | Trego | 2020 | 650465 |
| 20GO | Gove | 2020 | 1796859 |
| 20SC2 | Scott | 2020 | 762741 |
| 20WH | Wichita | 2020 | 60378 |
| 20JW3 | Jewell | 2020 | 324793 |
| 20MC2 | Mitchell | 2020 | 327715 |
| 20OB2 | Osborne | 2020 | 315507 |
| 20PL2 | Phillips | 2020 | 161659 |
| 20RP3 | Republic | 2020 | 361837 |
| 20KE2 | Kearny | 2020 | 129680 |
| 20KM | Kingman | 2020 | 367816 |
| 20SM3 | Smith | 2020 | 1392535 |
| 20SM4 | Smith | 2020 | 79933 |
