# Supplementary material for: Brome mosaic virus detected in Kansas wheat co-infected with other common wheat viruses
Source: Front Plant Sci. 2023 Mar 3;14:1096249. doi: 10.3389/fpls.2023.1096249 (PMC10022736; doi:10.3389/fpls.2023.1096249)
Supplement: Supplementary file 8 [file Table_4.docx]

Supplementary Table 4. A list of complete viral genome sequences and characterization of the consensus sequences of brome mosaic virus identified on wheat samples using Nanopore sequencing

| Sample ID | county | Accession number | Genome | No of reads† | Coverage (%) |
| --- | --- | --- | --- | --- | --- |
| 20SM3_BMV_RNA3^*^ | Smith | OP823149 | RNA3 | 942377 | 243202.96 |
| 20SM3_BMV_RNA2^θ^ | Smith | OP823153 | RNA2 | 64478 | 6437.2 |
| 20SM3_BMV_RNA1^#^ | Smith | OP823151 | RNA1 | 30245 | 3126.65 |
| 19CN1_BMV_MP^δ^ | Cheyenne | OP823155 | RNA3 | 236 | 100.72 |
| 19CN3 _BMV_MP ^δ^ | Cheyenne | OP823156 | RNA3 | 92 | 40.65 |
| 19DC1_BMV_MP^δ^ | Decatur | OP823157 | RNA3 | 241 | 113.76 |
| 19NS2_BMV_MP ^δ^ | Ness | OP823158 | RNA3 | 51 | 20.14 |
| 19JW1_BMV_MP ^δ^ | Jewell | OP823159 | RNA3 | 4597 | 1413.4 |
| 19JW1_BMV_CP^δ^ | Jewell | OP823160 | RNA3 | 4597 | 1413.4 |
| 19RP1_BMV_RNA3^*^ | Republic | OP823150 | RNA3 | 522697 | 191560.5 |
| 19RP1_BMV_RNA2^θ^ | Republic | OP823154 | RNA2 | 7513 | 1274.27 |
| 19RP1_BMV_RNA1^#^ | Republic | OP823152 | RNA1 | 4731 | 598.79 |

† Number of reads obtained using nanopore sequencing and mapped with reference genome using CLC Genomics Workbench

*Complete RNA3 sequence with both movement protein and coat protein-coding and non-coding regions

^δ^Incomplete RNA3 sequence with only movement protein-coding region

^θ^Complete RNA2 sequence with coding and non-coding regions

^#^ Complete RNA1 sequence with coding and non-coding regions
